# Disruption of the nuclear localization signal in RBM20 is causative in dilated cardiomyopathy

**DOI:** 10.1172/jci.insight.170001

**Published:** 2023-07-10

**Authors:** Yanghai Zhang, Zachery R. Gregorich, Yujuan Wang, Camila Urbano Braz, Jibin Zhang, Yang Liu, Peiheng Liu, Jiaxi Shen, Nanyumuzi Aori, Timothy A. Hacker, Henk Granzier, Wei Guo

**Affiliations:** 1 Department of Animal and Dairy Sciences, University of Wisconsin-Madison, Madison, Wisconsin, USA.; 2 Department of Animal Sciences, University of Illinois Urbana-Champaign, Urbana, Illinois, USA.; 3 Department of Anatomic Pathology, Comprehensive Cancer Center, City of Hope, Duarte, California, USA.; 4 Department of Integrative Biology,; 5 Department of Kinesiology, and; 6 Department of Psychology, University of Wisconsin-Madison, Madison, Wisconsin, USA.; 7 Division of Cardiovascular Medicine, Department of Medicine, School of Medicine and Public Health, University of Wisconsin-Madison, Madison, Wisconsin, USA.; 8 Department of Cellular and Molecular Medicine, University of Arizona, Tucson, Arizona, USA.; 9 Cardiovascular Research Center, School of Medicine and Public Health, University of Wisconsin-Madison, Madison, Wisconsin, USA.

**Keywords:** Cardiology, Cell Biology, Heart failure, Protein traffic, RNA processing

## Abstract

Human patients carrying genetic mutations in RNA binding motif 20 (RBM20) develop a clinically aggressive dilated cardiomyopathy (DCM). Genetic mutation knockin (KI) animal models imply that altered function of the arginine-serine-rich (RS) domain is crucial for severe DCM. To test this hypothesis, we generated an RS domain deletion mouse model (*Rbm20*^ΔRS^). We showed that *Rbm20*^ΔRS^ mice manifested DCM with mis-splicing of RBM20 target transcripts. We found that RBM20 was mis-localized to the sarcoplasm in *Rbm20*^ΔRS^ mouse hearts and formed RBM20 granules similar to those detected in mutation KI animals. In contrast, mice lacking the RNA recognition motif showed similar mis-splicing of major RBM20 target genes but did not develop DCM or exhibit RBM20 granule formation. Using in vitro studies with immunocytochemical staining, we demonstrated that only DCM-associated mutations in the RS domain facilitated RBM20 nucleocytoplasmic transport and promoted granule assembly. Further, we defined the core nuclear localization signal (NLS) within the RS domain of RBM20. Mutation analysis of phosphorylation sites in the RS domain suggested that this modification may be dispensable for RBM20 nucleocytoplasmic transport. Collectively, our findings revealed that disruption of RS domain–mediated nuclear localization is crucial for severe DCM caused by NLS mutations.

## Graphical abstract

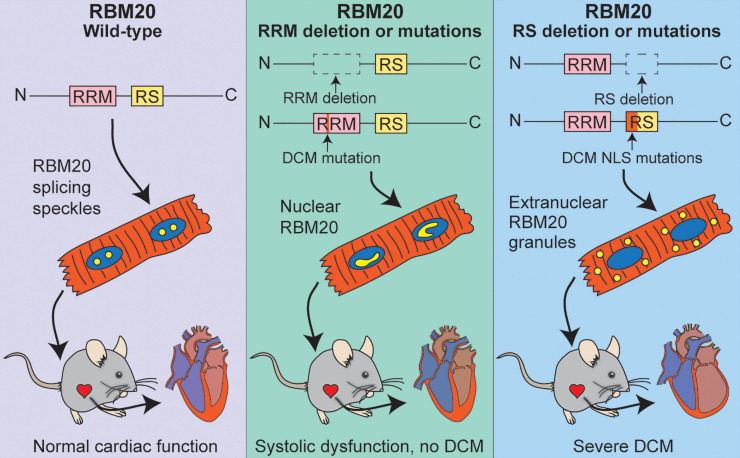

## Introduction

RNA binding motif 20 (RBM20) cardiomyopathy is a rare heart muscle disease caused by autosomal dominant mutations in the *RBM20* gene (1). RBM20 cardiomyopathy accounts for approximately 3% of familial dilated cardiomyopathy (DCM) cases (2, 3), with an increasing number of DCM-associated mutations having been identified (1, 4–20). RBM20-knockout rats and mice developed a DCM-like phenotype characterized by dilation of the left ventricle (LV), fibrosis, and an increased susceptibility to arrhythmia (21, 22). It was originally proposed that this phenotype arises because of the mis-splicing of RBM20 target genes (21, 22).

This splicing-centric view was challenged when mice expressing RBM20 lacking the RNA recognition motif (RRM) (*Rbm20*^ΔRRM^) were found to exhibit disrupted splicing of major RBM20 target genes and systolic dysfunction in the absence of cardiomyopathy (23). In contrast, RBM20 genetic mutations in human patients have been linked to aggressive DCM (1, 4, 22, 24). This linkage has now been validated in mutation knockin (KI) pig and mouse models (25–29). RBM20 R636S-KI pigs show severe DCM phenotype with high mortality at young age (28) similar to R636Q, S637A, and S639G mutation–KI (analogous to R634Q, S635A, and S637G in humans, respectively) mouse models (25–27, 29). All 4 of these mutations are in the arginine-serine-rich (RS) domain in RBM20. Recently, mice harboring the I538T variant (analogous to I536T in humans) located within the RRM in RBM20 were generated and were shown to develop neither DCM nor cardiac dysfunction despite altered splicing of RBM20 target transcripts (30). These findings led us to hypothesize that disruption of RS, rather than RRM, domain function is causative for RBM20 cardiomyopathy caused by RS domain mutations.

To test this hypothesis, we generated mice lacking the RS domain and characterized these mice using in vivo assessment of cardiac function, histology, immunohistochemical staining, and transcriptomic and posttranscriptomic analyses. Further, we defined the nuclear localization signal (NLS) located in the RS domain through in vitro experiments employing a series of sequence element deletion plasmids. We then determined whether mutations in other domains/regions of RBM20 facilitate RBM20 nucleocytoplasmic transport and granule assembly using in vitro cell culture and immunocytochemical staining. The necessity of RBM20 RS domain phosphorylation and sequence integrity for nuclear import was also evaluated using these techniques.

## Results

### Generation and characterization of Rbm20^ΔRS^ mice.

**Figure 1:**
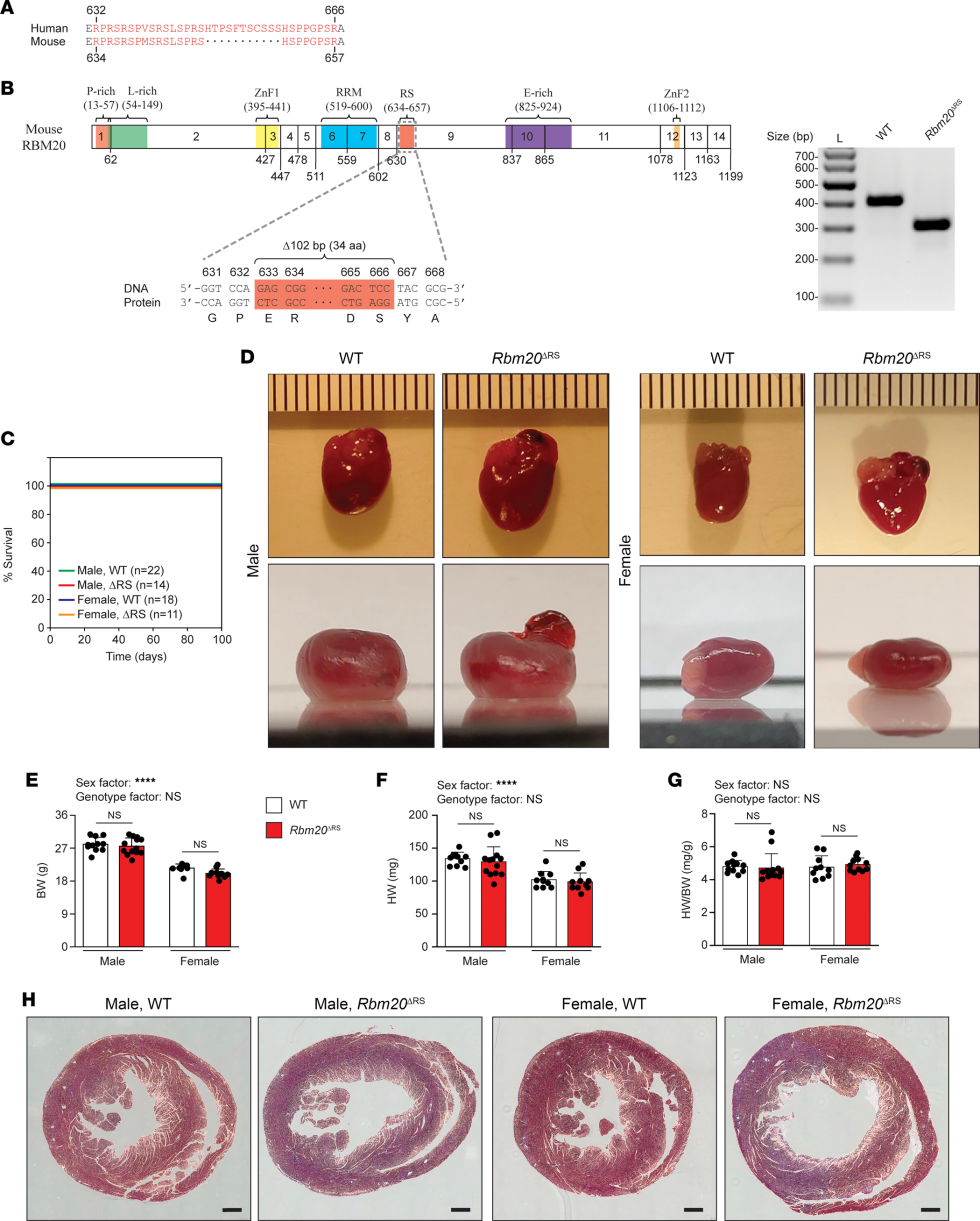
Characterization of male and female *Rbm20*^ΔRS^ mice. (**A**) Sequence alignment showing the sequence of the RS domain in human RBM20 and the corresponding sequence in mouse (amino acids 634–657). (**B**) Schematic showing the domain structure of mouse RBM20 and genetic manipulation to produce *Rbm20*^ΔRS^ mice. A 102 bp stretch was removed using CRISPR/Cas9 genome editing resulting in RBM20 lacking amino acid residues 633–666. Agarose gel confirming 102 bp deletion in the *Rbm20* gene in *Rbm20*^ΔRS^ mice is shown. (**C**) Kaplan-Meier survival curves for male and female WT and *Rbm20*^ΔRS^ mice. (**D**) Gross morphological characterization of hearts from 4-month-old male and female WT and *Rbm20*^ΔRS^ mice. Ruler spacing is 0.1 cm between ticks. (**E**–**G**) Bar graphs showing BW (**E**), HW (**F**), and HW/BW ratios (**G**) for 4-month-old male and female WT and *Rbm20*^ΔRS^ mice. Data are shown as mean ± SD. Dots represent measurements from individual animals. *n* = 11 and *n* = 10 for male WT and *Rbm20*^ΔRS^ mice, respectively. *n* = 10 and *n* = 11 for female WT and *Rbm20*^ΔRS^ mice, respectively. Two-way ANOVA with the Šídák method for multiple comparisons was performed to analyze the effect of sex and genotype on each of the aforementioned parameters. The interaction between sex and genotype was not significant for any parameter. ****P < 0.0001. (**H**) Representative Masson’s trichrome–stained heart sections from 2-month-old male and female WT and *Rbm20*^ΔRS^ mice. At least 3 heart sections were assessed per genotype per sex. Scale bars are 500 μm.

To determine whether disruption of RS domain function precipitates DCM, we generated mice expressing RBM20 lacking this domain. In humans, the RBM20 RS domain extends from amino acid residue 632 to 666 (21), which corresponds to amino acids 634–657 in the mouse (Figure 1A). Using CRISPR/Cas9 genome editing, the sequence corresponding to amino acid residues 633–666 in mouse RBM20 was deleted in-frame to generate *Rbm20*^ΔRS^ mice (Figure 1B). Deletion of the target sequence (102 bp) in *Rbm20*^ΔRS^ mice was confirmed by PCR and deep sequencing using an Illumina MiSeq System (Figure 1B and Supplemental Figure 1; supplemental material available online with this article; https://doi.org/10.1172/jci.insight.170001DS1). *Rbm20*^ΔRS^ mice were viable, fertile, and born at expected Mendelian and sex ratios. Previously described mouse lines carrying pathogenic RBM20 mutations exhibit significant mortality within the first 100 days after birth (25, 26, 29). In contrast to these previously described mouse lines, premature mortality was delayed in male and female *Rbm20*^ΔRS^ mice (Figure 1C). We did observe that the stress of pregnancy increased mortality in female *Rbm20*^ΔRS^ mice (Supplemental Figure 2). In general, hearts from 4-month-old *Rbm20*^ΔRS^ mice of both sexes were similar in size to those of age- and sex-matched WT controls but lacked the structural integrity of WT hearts, appearing flimsy and deflated by comparison (Figure 1D). Body weight (BW), heart weight (HW), and HW/BW ratios did not differ significantly between male and female *Rbm20*^ΔRS^ mice and age-matched WT controls of the same sex (Figure 1, E–G). Histological examination revealed dilation of the LV in both male and female *Rbm20*^ΔRS^ mice by 2 months of age (Figure 1H). There was a trend toward increased fibrosis in the hearts of 4-month-old female, but not male, *Rbm20*^ΔRS^ mice relative to that in WT mice of the same age and sex (Supplemental Figure 3).

### Rbm20^ΔRS^ mice develop a DCM-like phenotype characterized by chamber dilation and systolic dysfunction.

**Figure 2:**
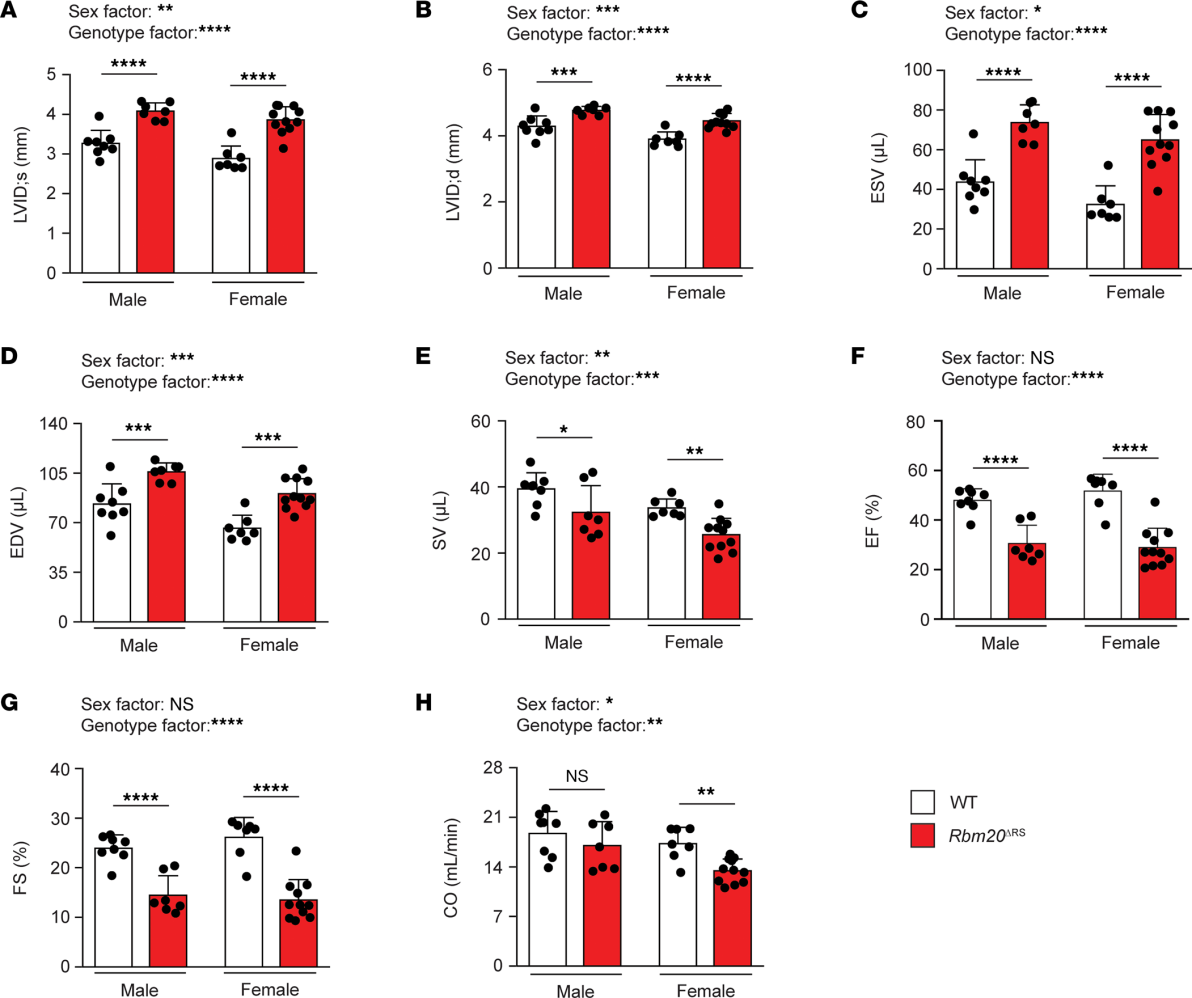
Male and female *Rbm20*^ΔRS^ mice develop a DCM-like phenotype. (**A**–**H**) Graphs showing LVID;s (**A**), LVID;d (**B**), ESV (**C**), EDV (**D**), SV (**E**), EF (**F**), FS (**G**), and CO (**H**) as determined by M-mode echocardiography in 4-month-old male WT (*n* = 8) and *Rbm20*^ΔRS^ (*n* = 7) mice, as well as female WT (*n* = 7) and *Rbm20*^ΔRS^ (*n* = 11) mice. Data are shown as mean ± SD. Dots represent measurements from individual animals. Two-way ANOVA with the Šídák method for multiple comparisons was performed to analyze the effect of sex and genotype on each of the aforementioned parameters. The interaction between sex and genotype was not significant for any parameter. *P < 0.05; **P < 0.01; ***P < 0.001; ****P < 0.0001.

To evaluate the effects of RBM20 RS domain deletion on cardiac function, male and female *Rbm20*^ΔRS^ mice were evaluated by noninvasive echocardiography at 4 months of age. Consistent with histological examination, echocardiography verified that the inner diameter of the LV was significantly increased at the end of systole (LVID;s) and diastole (LVID;d) in *Rbm20*^ΔRS^ mice of both sexes compared with age-matched WT controls of the same sex (Figure 2, A and B, and Supplemental Table 3). In line with this, the end systolic (ESV) and diastolic (EDV) volumes were significantly increased in the hearts of *Rbm20*^ΔRS^ mice of both sexes (Figure 2, C and D, and Supplemental Table 3). Systolic function was impaired in both male and female *Rbm20*^ΔRS^ mice, which had significantly reduced stroke volumes (SVs), ejection fractions (EFs), and fractional shortening (FS) compared with age- and sex-matched WT controls (Figure 2, E–G, and Supplemental Table 3). Cardiac output (CO) was significantly decreased in female, but not male, *Rbm20*^ΔRS^ mice in comparison with same-sex WT controls (Figure 2H and Supplemental Table 3). Taken together, these results demonstrate that *Rbm20*^ΔRS^ mice of both sexes develop a DCM-like phenotype (Figure 2 and Supplemental Table 3).

### Target gene splicing and expression are altered in the hearts of Rbm20^ΔRS^ mice.

**Figure 3:**
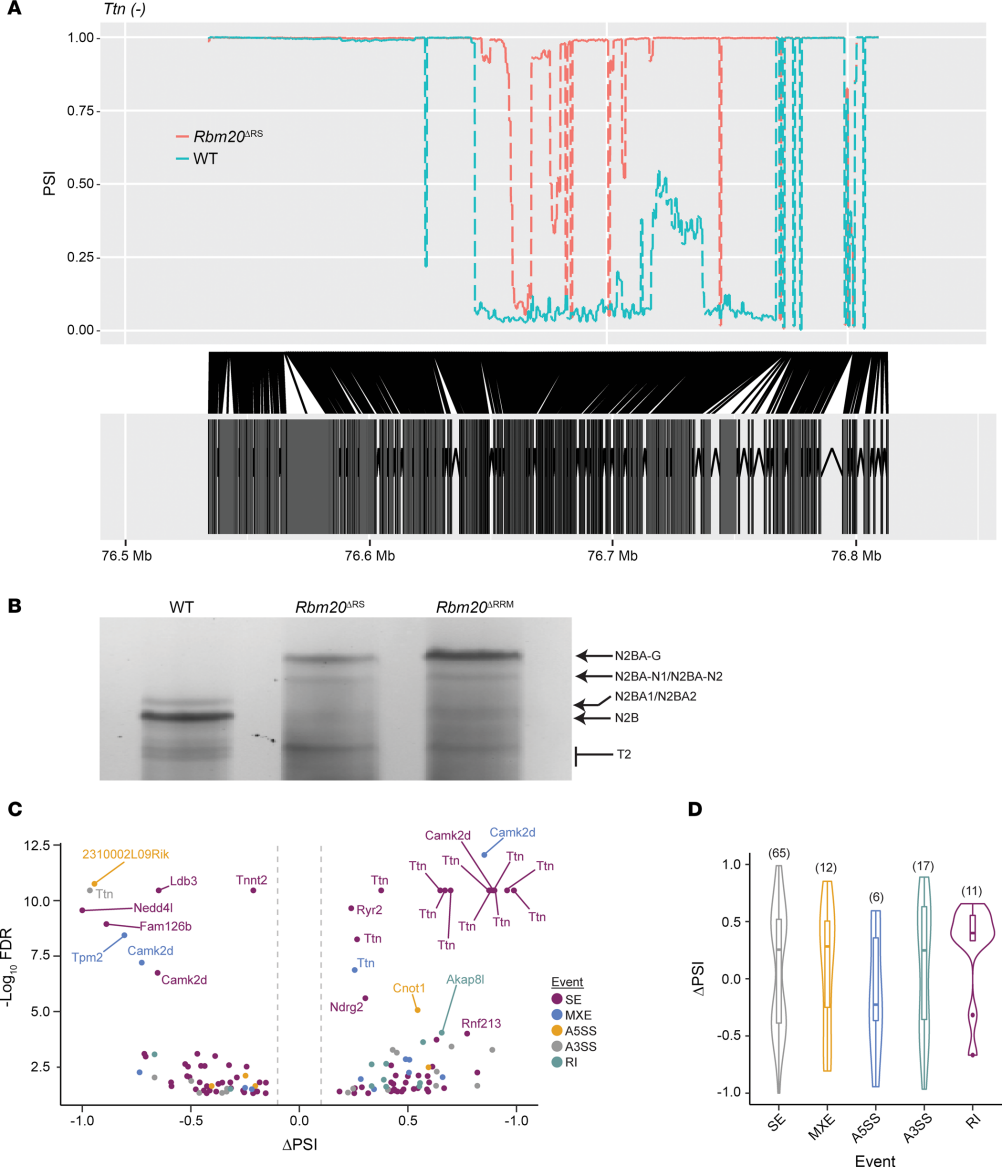
Splicing of RBM20 target genes is disrupted in the hearts of male *Rbm20*^ΔRS^ mice. (**A**) RNA-Seq percentage spliced in (PSI) alternative splicing maps for *Ttn* comparing WT (blue) and *Rbm20*^ΔRS^ (red). (**B**) Titin isoforms detected in the LVs of WT, *Rbm20*^ΔRS^, and *Rbm20*^ΔRRM^ mice. Data are from a single experiment. (**C**) Volcano plot showing genes that are differentially spliced in the hearts of *Rbm20*^ΔRS^ mice relative to WT control (data are from *n* = 3 per genotype). Genes with −log_10_ FDR > 5 and |ΔPSI| > 0.1 are labeled. SE, skipped exon; MXE, mutually exclusive exon; A5SS, alternative 5′ splice site; A3SS, alternative 3′ splice site; RI, retained intron. (**D**) Violin plots representing the distributions of statistically significant ΔPSI values for the different classes of AS events in *Rbm20*^ΔRS^ mice relative to WT controls: ΔPSI = PSI(ΔRS) − PSI(WT) (data are from *n* = 3 per genotype). The lower and upper bounds of the embedded box represented the 25th and 75th percentile of the distribution, respectively. The horizontal line in the box represented the median. The numbers of events are shown above each plot.

Disrupted splicing of RBM20 target genes is characteristic of RBM20 cardiomyopathy (25–28). To determine whether splicing of RBM20 targets is also disrupted in the hearts of *Rbm20*^ΔRS^ mice, RNA was extracted from the LVs of 2-month-old male WT and *Rbm20*^ΔRS^ mice, then submitted for RNA-Seq, and differentially spliced genes (DSGs) were identified. A total of 103 genes were differentially spliced in the hearts of *Rbm20*^ΔRS^ mice in comparison with WT controls, including several well-established RBM20 splicing targets such as *Ttn*, *Camk2d*, *Ryr2*, and *Tpm2* (Figure 3A and Supplemental Table 4) (21, 31). To validate disrupted splicing of *Ttn*, which is the primary splice target of RBM20 (21), proteins were extracted from the ventricular myocardium of 2-month-old male *Rbm20*^ΔRS^ mice, and the expression of titin isoforms was assessed by agarose gel electrophoresis. Extracts prepared from age-matched male WT and *Rbm20*^ΔRRM^ mice served as negative and positive controls, respectively, for disrupted *Ttn* splicing (23). N2B titin was the predominant isoform expressed in the ventricle of age-matched WT mice (Figure 3B). On the other hand, titin was shifted to a giant N2BA (N2BA-G) isoform in *Rbm20*^ΔRS^ mice like that observed in *Rbm20*^ΔRRM^ mice (Figure 3B) (23), validating that *Ttn* splicing is impaired in the hearts of these mice. All 5 alternative splicing events were represented among the DSGs, including 65 events with skipped exons (SEs), 6 with an alternative 5′ splice site (A5SS), 17 with an alternative 3′ splice site (A3SS), 12 with mutually exclusive exons (MXEs), and 11 with retained introns (RIs) (Figure 3, C and D). The differential splicing of 3 other RBM20 target genes, *Camk2d*, *Ryr2*, and *Tpm2*, was also validated by reverse transcription PCR (RT-PCR), and results were in line with the RNA-Seq results (Supplemental Figure 4). Thus, *Rbm20*^ΔRS^ mice are deficient in RBM20 splicing.

In addition to DSGs, 1,399 genes were found to be differentially expressed in the hearts of *Rbm20*^ΔRS^ mice relative to expression in age-matched WT controls (Supplemental Figure 5 and Supplemental Table 5). Of the 1,399 genes, 575 and 824 were significantly up- and downregulated, respectively (Supplemental Figure 5 and Supplemental Table 5). We validated changes in the expression of several of these genes, including *Nppa*, *Kcne1*, *Ankrd1*, *Ccn2*, *Fbn2*, *Scn4b*, *Fah*, and *Wnt5a*, with RT-qPCR (Supplemental Figure 6). In particular, we validated upregulation of the cardiac stress/heart failure genes *Nppa* and *Ankrd1* (32, 33) in the hearts of *Rbm20*^ΔRS^ mice (Supplemental Figure 6). In addition, we examined 2 potential partners of RBM20, PTBP1 and U2AF65 (21, 31, 34), to determine whether deletion of the RS domain affected their expression. Western blot analysis revealed that the expression of these proteins was unchanged in the ventricular myocardium of *Rbm20*^ΔRS^ mice (Supplemental Figure 7). Collectively, these results support the development of cardiac stress and failure in these mice and demonstrate that the expression of the RBM20 partners PTBP1 and U2AF65 are not affected by loss of the RS domain.

### RBM20 is mis-localized in the hearts of Rbm20^ΔRS^ mice.

**Figure 4:**
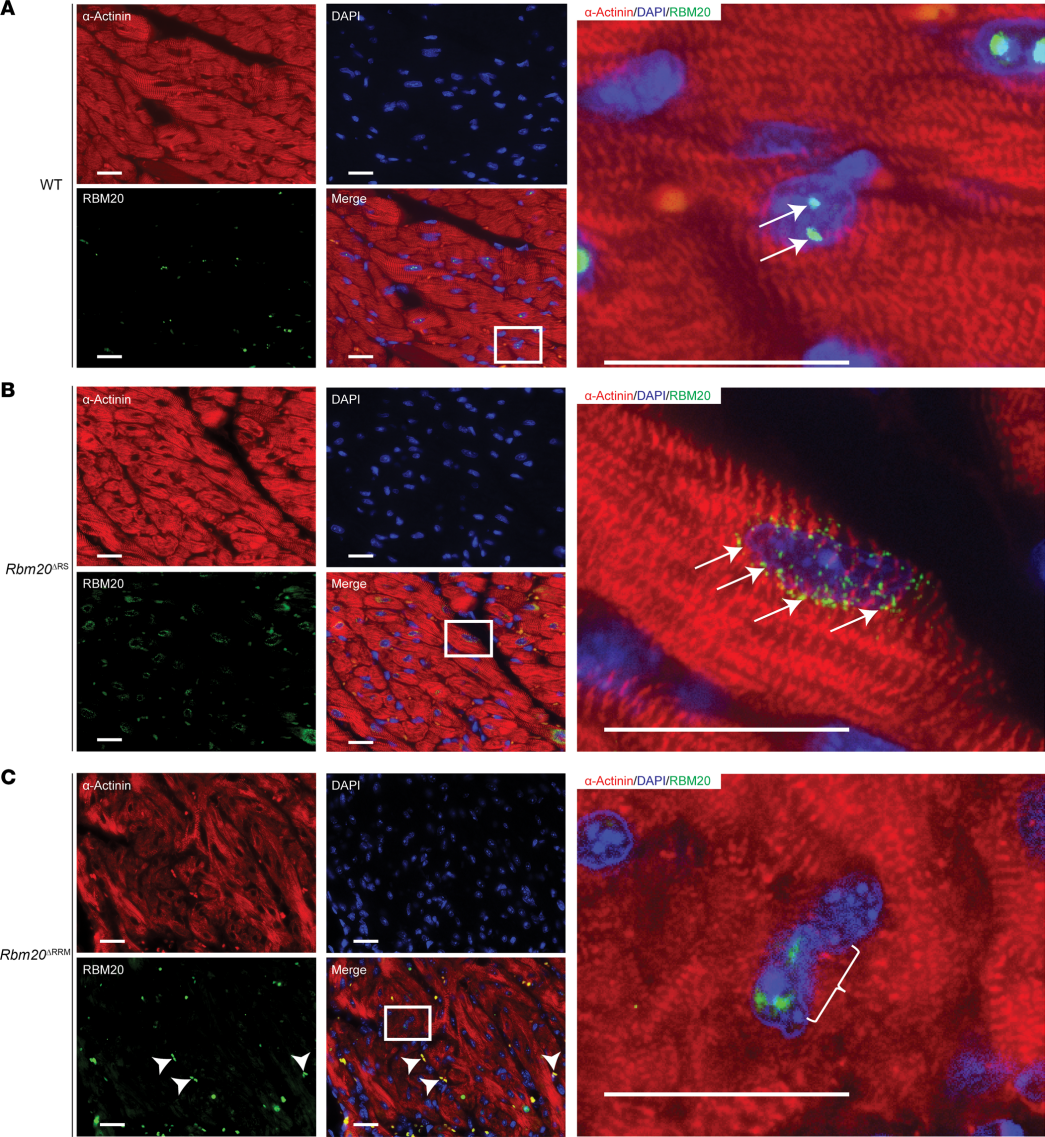
Loss of the RS, but not RRM, domain in RBM20 promotes sarcoplasmic localization in male mouse hearts. (**A**–**C**) Representative IHC images showing the localization of RBM20 in the hearts of male WT (**A**), *Rbm20*^ΔRS^ (**B**), and *Rbm20*^ΔRRM^ (**C**) mice. Sections from 3 animals per genotype were assessed. Inset images are shown on the right. Arrows and bracket denote RBM20 staining. Arrowheads mark nonspecific staining of noncardiomyocytes. Scale bars are 25 μm.

Recently, our lab and others have shown that pathogenic mutations in the RBM20 RS domain promote nucleocytoplasmic shuttling and sarcoplasmic accumulation of the protein (14, 25–29). Thus, we hypothesized that deletion of the RS domain in RBM20 prevents nuclear import and/or retention of the protein in the nucleus. To test this hypothesis, the localization of RBM20 was determined by IHC in cardiac sections from 2-month-old male WT and *Rbm20*^ΔRS^ mice. In age-matched male WT mice, RBM20 was localized to 2 discrete speckles in the nucleus previously shown to serve as sites for *Ttn* pre-mRNA processing (arrows, Figure 4A, right; and Supplemental Video 1) (35). Consistent with our hypothesis, in *Rbm20*^ΔRS^ mice RBM20 was localized to perinuclear puncta resembling the RBM20 granules previously reported in humans and other animals carrying RBM20 missense mutations (arrows, Figure 4B, right; and Supplemental Video 2) (14, 25–29). The localization of RBM20 was also assessed in the hearts of 2-month-old male *Rbm20*^ΔRRM^ mice. Unlike in *Rbm20*^ΔRS^ mice, RBM20 lacking the RRM was localized exclusively within the nucleus (bracket, Figure 4C, right; and Supplemental Video 3). These findings indicate that the RS domain plays a critical role in RBM20 nuclear localization.

### The RS domain of RBM20 mediates nuclear localization.

**Figure 5:**
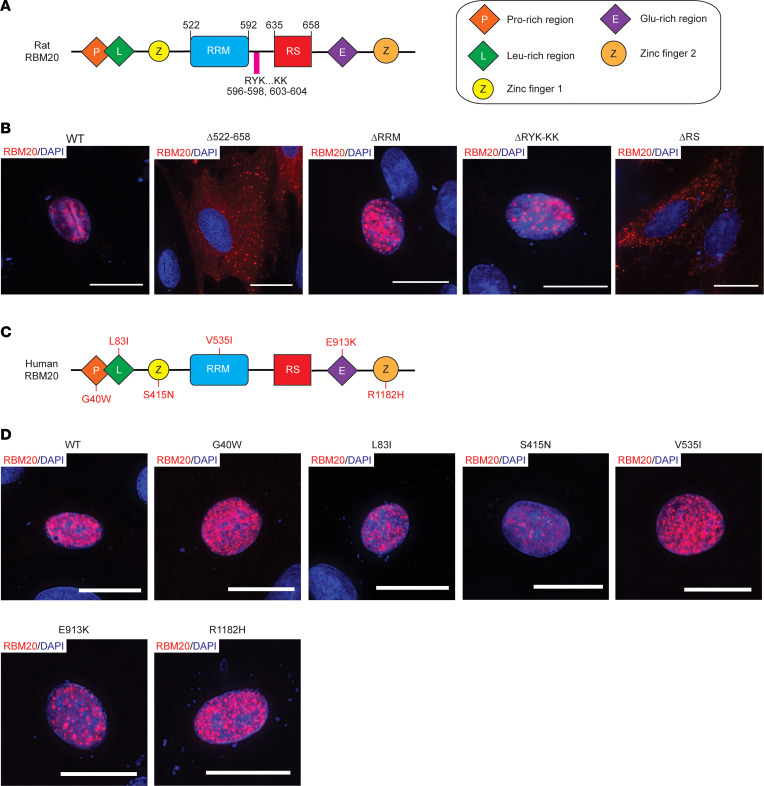
Deletion of the RS domain, but not the RRM, disrupts RBM20 nuclear localization. (**A**) Schematic showing domain structure of rat RBM20. (**B**) Representative immunocytochemistry (ICC) images showing rat RBM20 domain and sequence deletion construct localization in transfected H9c2 cells. (**C**) Schematic showing domain structure of human RBM20 with DCM-associated mutations in the respective domains/regions within the protein listed. (**D**) Representative ICC images showing the localization of human RBM20 constructs harboring DCM-associated mutations in domains/regions other than the RS domain expressed in H9c2 cells. Scale bars are 20 μm. All transfection experiments were repeated at least once for a total of 2 replicates.

To verify that the RS domain mediates RBM20 nuclear localization, we generated rat RBM20 constructs containing WT RBM20, RBM20 lacking the entire region from the start of the RRM domain to the end of the RS domain (Δ522–658), RBM20 lacking the RRM domain (ΔRRM), RBM20 lacking a classical monopartite NLS (KRYK) located between the RRM and RS domains that has been described previously (ΔRYK-KK) (36), and RBM20 lacking the RS domain (ΔRS) (Figure 5A).

To determine the role of these domains and sequence elements in mediating RBM20 nuclear localization, H9c2 cells were transfected with the different constructs, and the localization of RBM20 was demonstrated by ICC. As expected, WT RBM20 was exclusively localized within the nucleus of transfected cells (Figure 5B). Conversely, the Δ522–658 construct localized to the cytoplasm of transfected H9c2 cells (Figure 5B) (37). Unlike the Δ522–658 construct, both the ΔRRM and ΔRYK-KK constructs were contained within the nucleus (Figure 5B), indicating that these sequences are not necessary for nuclear localization. Localization of the ΔRRM construct to the nucleus of transfected H9c2 agreed with the localization of RBM20 in the hearts of *Rbm20*^ΔRRM^ mice (Figure 4C). In contrast, the ΔRS construct localized to the cytoplasm in transfected cells (Figure 5B), which is consistent with the sarcoplasmic accumulation of RBM20 observed in *Rbm20*^ΔRS^ mouse hearts (Figure 4B).

The RS domain of RBM20 is 1 of 2 DCM-associated mutation hotspots in the protein, with the other being the glutamate-rich region (38). Notably, a growing number of mutations in other domains/regions of the protein have also been reported in the context of DCM. A number of mutations in the RS domain have been shown to impair RBM20 nuclear localization (14, 25–29). To verify that DCM-associated mutations in other domains/regions outside the RS domain do not impair nuclear localization of the protein, we engineered the G40W (20), L83I (5), S415N, V535I (4), E913K (9), and R1182H (8, 20) mutations into the respective domains/regions of human RBM20, and the localization of these constructs was determined by ICC in transfected H9c2 cells (Figure 5C). It should be noted that, although the S415N mutation has not been reported in the literature, according to information in the ClinVar database (ClinVar accession: VCV000548140.4), this variant was discovered in a woman with early-onset DCM and heart failure. Consistent with the dominant role played by the RS domain in determining RBM20 nuclear localization, all mutant constructs were confined to the nucleus of transfected cells (Figure 5D). Collectively, these results convincingly demonstrate that only the RS domain of RBM20 is responsible for mediating nuclear localization of the protein.

### The D1 sequence element constitutes the core NLS in RBM20.

**Figure 6:**
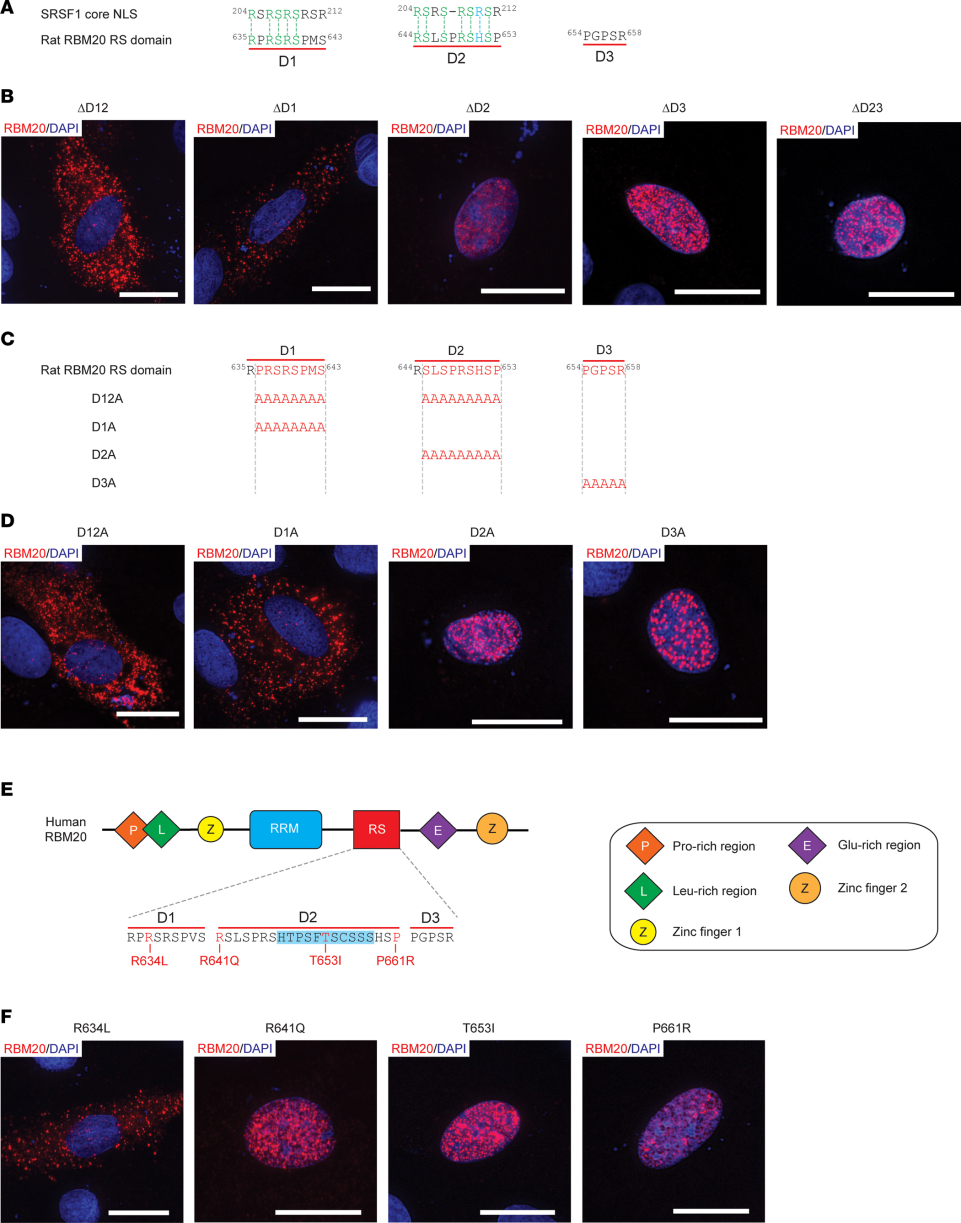
Disruption of the D1 core NLS sequence element abolishes RBM20 nuclear localization. (**A**) In silico identification of SRSF1 core NLS-like sequence elements in the RS domain of rat RBM20. Conserved amino acids are shown in green and blue denotes a positively charged amino acid. (**B**) Representative ICC images showing rat RBM20 deletion construct localization in transfected H9c2 cells. (**C**) Schematic showing rat RBM20 Ala substitution constructs for in vitro expression in H9c2 cells. (**D**) Representative ICC images showing rat RBM20 Ala substitution construct localization in transfected H9c2 cells. Scale bars are 20 μm. (**E**) Schematic showing human RBM20 domain structure with a zoom-in on the RS domain sequence and DCM-associated mutations listed. The human-specific segment in the D2 sequence element is highlighted in light blue. (**F**) Representative ICC images showing the localization of human RBM20 constructs harboring DCM-associated mutations in the D1 and D2 sequence elements. Scale bars are 20 μm. All transfection experiments were repeated at least once for a total of 2 replicates.

To elucidate the mechanism underlying RS domain–mediated control of RBM20’s nuclear localization, in silico analysis was used to identify putative NLS sequence elements within the RBM20 RS domain using the core transportin-interacting SRSF1 RS domain sequence (i.e., RSRSRSRSR) (39) as a template. The core transportin-interacting SRSF1 RS domain sequence was chosen as a template because SRSF1 is the prototypical serine-arginine-rich (SR) protein, and interactions between this sequence and the nuclear import receptor *Tnpo3* have been studied previously (39). In silico analysis revealed the presence of 2 core SRSF1 RS domain sequence-like elements located within the RS domain of RBM20, hereafter referred to as D1 and D2 (Figure 6A). The remaining sequence in the RS domain not included in these 2 sequence elements is referred to as D3. To determine which of these sequence elements are required for RBM20 nuclear localization, we made rat RBM20 constructs lacking both D1 and D2 (ΔD12), D1 only (ΔD1), D2 only (ΔD2), D3 only (ΔD3), and both D2 and D3 (ΔD23). These constructs were transfected in H9c2 cells, and the localization of RBM20 was determined by ICC. Like the ΔD12 construct, the ΔD1 construct localized exclusively to the cytoplasm of H9c2 (Figure 6B). In contrast, the ΔD2, ΔD3, and ΔD23 constructs were all retained within the nucleus (Figure 6B).

Rat RBM20 mutant constructs with Ala residues in place of amino acids 636–643 in D1, 645–653 in D2, or 654–658 in D3 were generated to further validate the necessity of the D1 sequence element for nuclear import. The D12A construct contained Ala substitution for both D1 and D2 (Figure 6C). In agreement with the results of the previous deletion experiments, constructs in which the D1 sequence element was replaced with Ala residues (D1A and D12A) exhibited a cytoplasmic localization pattern while those containing an intact D1 sequence element (D2A and D3A) were located within the nucleus (Figure 6D).

In addition to the growing list of mutations reported in the D1 sequence element, several DCM-associated mutations have also been reported in D2, including variants in a stretch of amino acids not conserved in the D2 sequence element in rat RBM20 (sequence highlighted in blue, Figure 6E). To determine whether DCM-associated mutations in the human D2 sequence element promote RBM20 relocalization, the R641Q (20), T653I (40), and P661R (ClinVar accession: VCV000965264.3) mutations were engineered into human RBM20, and the mutant constructs were expressed in H9c2 cells. The R634L mutation (41), which is located in the D1 sequence element, served as a positive control for disrupted nuclear localization. In agreement with the D1/D2 deletion and Ala replacement experiments in rat RBM20 (Figure 6, A–D), only the R634L mutation, located in the D1 sequence element, promoted accumulation of human RBM20 in the cytoplasm of transfected H9c2 cells (Figure 6F). Collectively, these results validate that the D1 sequence element is the core NLS in RBM20 and suggest that mutations in the D1 core NLS alone promote severe RBM20 cardiomyopathy via impaired nuclear import and accumulation in cytoplasmic granules.

### Pseudo-phosphorylation of the RS domain of RBM20 does not rescue nuclear localization.

**Figure 7:**
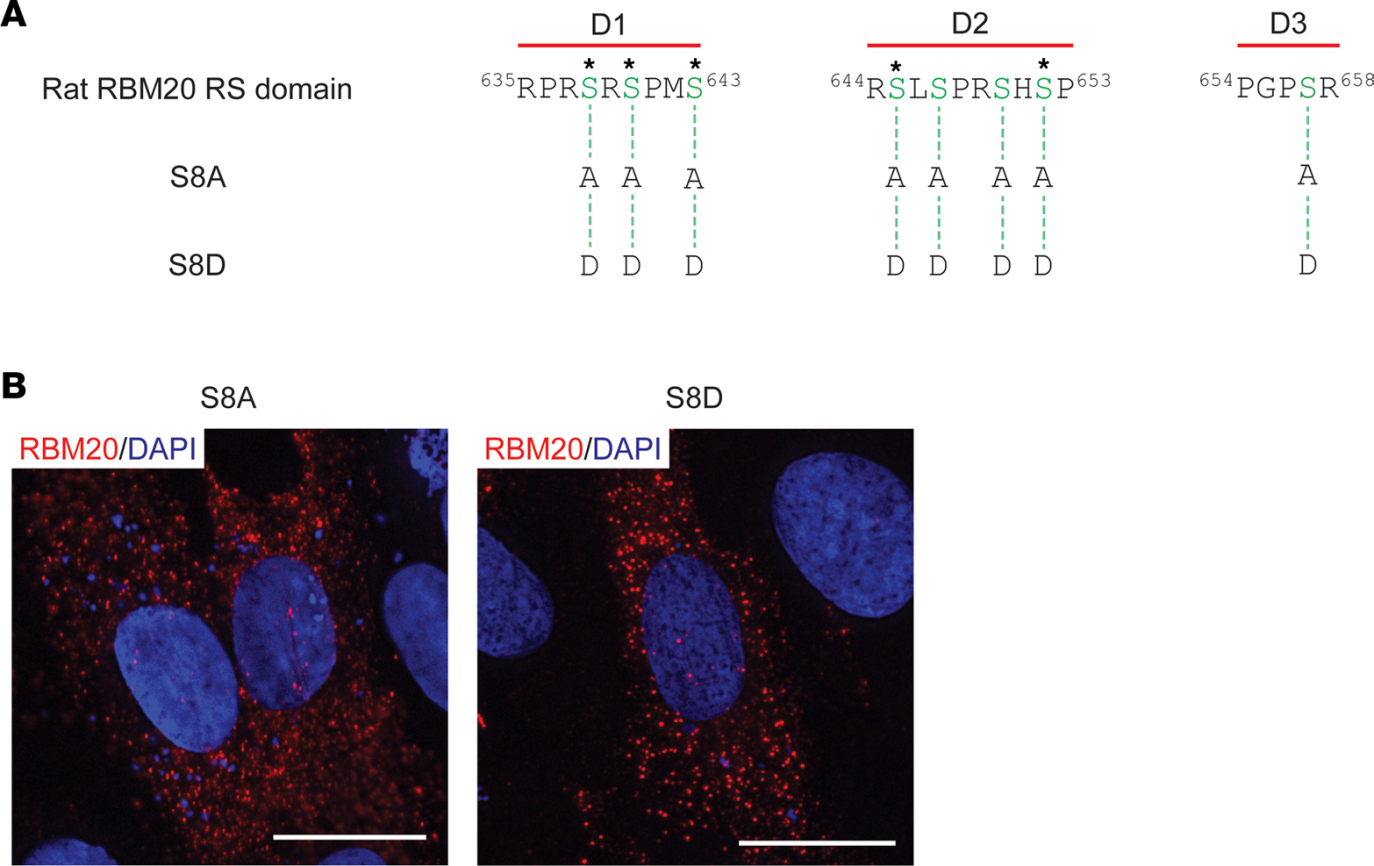
Replacement of phosphorylatable Ser residues in the RBM20 RS domain with phosphomimetic Asp residues does not rescue nuclear localization. (**A**) Schematic showing rat RBM20 Ala and Asp mutation constructs for in vitro expression in H9c2 cells. Asterisks denote Ser residues previously identified as phosphorylation sites (26, 42). (**B**) Representative ICC images showing RBM20 Ala and Asp mutation construct localization in transfected H9c2 cells. Scale bars are 20 μm. All transfection experiments were repeated at least once for a total of 2 replicates.

Recently, it was found that multiple Ser residues in the RS domain of RBM20, including all 3 Ser residues in the D1 sequence element, are basally phosphorylated (26, 42). Given that RS domain phosphorylation has previously been shown to be important for nucleocytoplasmic shuttling of SR proteins such as SRSF1 (43, 44), we next sought to establish the role of RS domain phosphorylation in RBM20 nuclear localization. Constructs were generated in which all phosphorylatable Ser residues within the RS domain were mutated to either nonphosphorylatable Ala (S8A) residues or phosphomimetic Asp residues (S8D) (Figure 7A). Mutation to Ala residues prevented RBM20 nuclear import (Figure 7B). Conversely, mutation to the phosphomimetic residue Asp did not rescue nuclear localization of RBM20 (Figure 7B). Although these results suggest that phosphorylation of the NLS in RBM20 may not be required for nuclear localization, this evidence alone is not strong enough to support this conclusion.

### D1 amino acid composition is critical for RBM20 nuclear localization.

**Figure 8:**
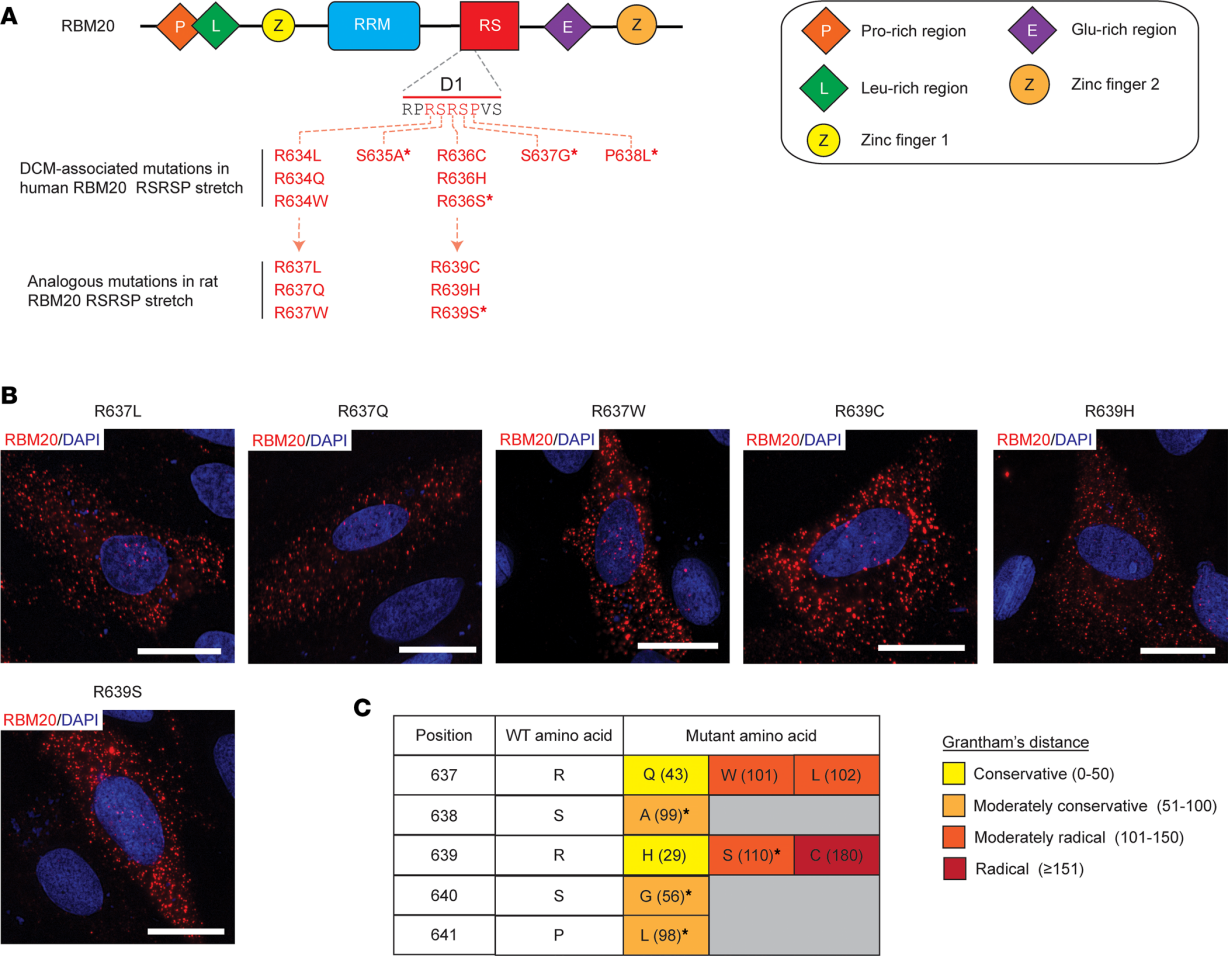
DCM-linked mutations in the RSRSP stretch contained within the D1 sequence element disrupt RBM20 nuclear localization. (**A**) Schematic showing the domain structure of human RBM20 with the D1 sequence element shown. Critical RSRSP stretch is highlighted in red. Reported mutations in the RSRSP stretch in human RBM20 are listed along with the corresponding mutations in rat RBM20. Asterisks denote RBM20 mutants with localization data already available in the literature. (**B**) Representative images showing the localization of rat RBM20 harboring DCM-linked mutations corresponding those reported in the RSRSP stretch of human RBM20. Scale bars are 20 μm. All transfection experiments were repeated at least once for a total of 2 replicates. (**C**) Table displaying Grantham’s distances for DCM-linked amino acid mutations in the RSRSP stretch that disrupt RBM20 nuclear localization. Amino acid positions are given for rat RBM20. The number in parentheses corresponds to Grantham’s distance between the amino acid in the WT sequence and the mutant amino acid. Grantham’s distances were classified as conservative, moderately conservative, moderately radical, or radical as proposed by Li et al. (46). Asterisks denote RBM20 mutants with localization data already available in the literature.

To assess the role that D1 amino acid composition plays in mediating RBM20 nuclear localization, we examined the localization of RBM20 constructs harboring 5 DCM-associated NLS mutations for which no available localization data have been reported to our knowledge. The selected mutations included R634L, R634Q, R634W, R636C, and R636H (analogous to R637L, R637Q, R637W, R639C, and R639H in rat, respectively) and were chosen as they represent amino acid substitutions with differing severity (Figure 8A). For example, considering Grantham’s distances (d), which provides a proxy for the interchangeability of 2 amino acids based on their composition, polarity, and molecular volume (45), R636H (d = 29; both residues are polar and basic, differing primarily in molecular volume, with His being smaller than Arg) is considered a conservative substitution while R636C (d = 180; both residues are polar, but Cys is uncharged and is smaller in terms of molecular volume) is a radical substitution based on the classification system established by Li et al. (46). The R636S mutation served as a positive control for disrupted nuclear localization (Figure 8A) (28, 37). The selected mutations were engineered into rat RBM20, and the subcellular localization of the mutant proteins was assessed following transfection in H9c2 cells using ICC. Consistent with previous reports (28, 37), rat RBM20 harboring the R639S mutation (analogous to R636S in human; d = 110, moderately radical substitution) exhibited a cytoplasmic localization pattern (Figure 8, B and C). Strikingly, all 5 of the selected mutations also disrupted the nuclear import of RBM20 and were localized to the cytoplasm regardless of the severity of the amino acid substitution (Figure 8, B and C). These results validate that the amino acid sequence of the D1 NLS is essential for RBM20 nuclear import.

## Discussion

There are 3 widely accepted mechanisms contributing to RBM20 cardiomyopathy. These are i) a reduction in titin-based passive stiffness resulting from changes in titin isoform expression (21, 23, 47), ii) impaired contractile function secondary to altered splicing of Ca^2+^-handling genes (22), and iii) pathophysiological gain-of-function effects caused by RBM20 nucleocytoplasmic shuttling and sarcoplasmic accumulation in RBM20 granules (14, 25–29). However, it remains unclear which of these mechanisms is the driving force behind the development of DCM. Notably, multiple studies have now shown that mice with disrupted splicing of RBM20 target transcripts do not develop an overt cardiomyopathy (23, 30). These findings indicate that altered splicing alone does not cause RBM20 cardiomyopathy. Herein, employing a potentially novel RBM20 RS domain deletion mouse model in combination with in vitro experiments, we show that RBM20 mis-localization, rather than disrupted splicing, is the driving mechanism in RBM20 cardiomyopathy caused by NLS mutations. Based on this finding, we hypothesize that RBM20 nucleocytoplasmic trafficking and accumulation in sarcoplasmic RBM20 granules underlies the development of RBM20 cardiomyopathy associated with NLS mutations, although disruption of an unknown nonsplicing nuclear function of RBM20 cannot be ruled out at this time. This hypothesis is supported by the fact that animals carrying RBM20 mutations that promote RBM20 granule formation in the sarcoplasm develop severe DCM (25–29). With the shift in mechanistic focus from mis-splicing to RBM20 granules as the causative mechanism in RBM20 cardiomyopathy caused by NLS mutations, an important next step will be to determine whether preventing nucleocytoplasmic trafficking of RBM20 by stabilizing the complex formed between importer proteins and the disrupted core NLS sequence or targeted disassembly of sarcoplasmic RBM20 granules can benefit this disease.

One question that remains unanswered is why the premature mortality observed in mice harboring NLS mutations in RBM20 (25, 26, 29) was delayed in *Rbm20*^ΔRS^ mice even though RBM20 granules appeared to be present. We believe that this may be because the RS domain plays an important role in determining disease severity by influencing the composition of RBM20 granules. Indeed, the RS domain in SR proteins is known to mediate a host of protein-protein interactions, including those essential for assembly of the spliceosome (48). Our present hypothesis is that residual RS domain function in the context of pathogenic mutations in RBM20 enables the recruitment of a specific complement of proteins and/or RNAs to RBM20 granules, leading to a more severe DCM phenotype. Nevertheless, additional studies will be necessary to validate this hypothesis.

Another important finding of the present study is that the RS domain, not the RRM, controls RBM20 nuclear localization. The findings of a previous study conducted by Filippello et al. suggested that additional sequences in RBM20, specifically the RRM and sequence between the RRM and RS domains, may be required for nuclear retention of the protein (36). Herein, we provide 2 lines of evidence indicating that the RS domain is solely responsible for RBM20 nuclear localization. First, we demonstrate that RBM20 nuclear localization is disrupted in the hearts of *Rbm20*^ΔRS^ mice. Second, follow-up experiments in transfected H9c2 cells showed that, while the RRM was dispensable for nuclear localization, the RS domain was necessary. This finding is relevant to the mechanism(s) underlying the development of DCM in RBM20 mutation carriers as it indicates that RBM20 nucleocytoplasmic transport is the driving force only in patients harboring mutations in the NLS within the RS domain. Consistently, our data show that DCM-associated mutations in domains other than the RS domains do not promote relocalization of RBM20 to the cytoplasm in transfected cells. Thus, mutations outside the NLS in the RS domain must cause DCM via another unknown mechanism unrelated to RBM20 nucleocytoplasmic transport and sarcoplasmic granule formation.

Consistent with the role for the RS domain in controlling RBM20 nuclear import, we identified the D1 sequence element as the core NLS in RBM20. This finding is supported by the observation that mutations in the RSRSP stretch, which is located within the D1 core NLS, promote nucleocytoplasmic transport and accumulation of the protein in the sarcoplasm (25–28). Surprisingly, while not all possible amino acid substitution permutations have been tested, here we show that even conservative amino acid substitutions, such as R634Q and R634H, within this sequence are sufficient to disrupt nuclear localization of the protein. Thus, the RS domain, specifically, the integrity of the D1 core NLS, is essential for nuclear import of RBM20. Identification of the nuclear import receptor for RBM20 will be a key step for understanding how mutations in this NLS disrupt nuclear import.

Prior studies have shown that phosphorylation of the RS domain in SRSF1, the prototypical SR protein, is also important for interactions with SR-specific transportin proteins and, thus, for nuclear import of the protein (39, 43, 44). In the case of RBM20, Murayama et al. demonstrated that mutation of 2 phosphorylated Ser residues (S637 and S639 in mice, or S635 and S637 in humans) in the RS domain of RBM20 disrupted splicing of a *Ttn* reporter and promoted nuclear exclusion of the protein (42). We have reported similar findings; yet, replacement of these 2 Ser residues with the phosphomimetic amino acid Asp, which has previously been shown to at least partially replace phosphorylated Ser with respect to mediating SR protein nuclear import (49), did not rescue nuclear localization (26). Importantly, we recently identified several additional sites of phosphorylation within the RS domain using middle-down mass spectrometry (26), raising the question of whether phosphorylation at other sites regulates RBM20 nuclear localization. In this study, we mutated all phosphorylatable Ser residues in the RS domain of rat RBM20 to either Ala or Asp residues. Surprisingly, replacement of Ser residues in the RS domain with phosphomimetic Asp residues did not restore nuclear localization of RBM20. Taken together, these results suggest that phosphorylation of the RBM20 RS domain may not be required for nuclear localization. However, a notable caveat is that, to date, all studies that have examined the role of RBM20 phosphorylation in nuclear localization have employed mutagenesis to prevent phosphorylation at specific sites in the protein (26, 42). Given that mutations themselves disrupt RBM20 localization, assessing the role phosphorylation plays in subcellular localization by methods other than mutagenesis will be a necessary future step for confirming these findings. Moreover, these experiments cannot exclude the possibility that phosphorylation at specific sites or combinations of sites is necessary for nuclear import or that mutations in the NLS impair nuclear localization by disrupting the phosphorylation of Ser residues in this sequence.

## Methods

### Experimental animals and tissue collection.

*Rbm20*^ΔRS^ mice were generated via CRISPR/Cas9 genome editing in collaboration with the Advanced Genome Editing Animal Models Core Facility at the University of Wisconsin-Madison by introducing Cas9 protein (6 μM, IDT v3), 2 sgRNAs (3.3 μM each, IDT sgRNAs), and a single-stranded donor (20 ng/μL) into C57BL/6J embryos by electroporation (sequence information for single-stranded donor and sgRNAs is provided in Supplemental Table 1). Founders were screened using Illumina targeted deep sequencing to identify animals carrying perfect excisions of the region E633-S666 in RBM20. Founders were backcrossed to C57BL/6J mice (strain 000664; The Jackson Laboratory) to generate F_1_ animals, which were similarly characterized by targeted deep sequencing. Mice were not backcrossed further. Heterozygous founders were crossed to produce homozygous *Rbm20*^ΔRS^ mice for subsequent characterization. For genotyping, genomic DNA was isolated from toe clips by incubating in DirectPCR (Tail) Lysis Reagent for Genotyping Crude Lysates (catalog 102-T; Viagen Biotech) with proteinase K (catalog DSP50220-0.1; Dot Scientific) for 12 hours at 55°C followed by a 45-minute heat inactivation at 85°C. Isolated genomic DNA was combined with EconoTaq PLUS GREEN 2X Master Mix (catalog 30033-1; Lucigen) and forward and reverse primers (primer information listed in Supplemental Table 2), and PCR was carried out with an initial denaturation at 94°C for 2 minutes, followed by 35 cycles of 94°C for 15 seconds, 63°C for 30 seconds, and 72°C for 30 seconds. *Rbm20*^ΔRRM^ mice are also in the C57BL/6J background and have been described previously (23). WT C57BL/6J mice served as controls for all experiments.

All mice were maintained on standard rodent chow (catalog 8604; Envigo) and were housed in a facility on a 12-hour light/12-hour dark cycle with access to food and water ad libitum. Hearts were collected from 2- and 4-month-old mice immediately following euthanasia. After removal, hearts were either paraffin-embedded for histology and/or immunohistochemistry (IHC) or sectioned into chambers, snap-frozen in liquid nitrogen, and stored in a −80°C freezer for later biochemical experiments.

### Histology.

Whole hearts from 2- or 4-month-old WT, *Rbm20*^ΔRS^, and *Rbm20*^ΔRRM^ mice were isolated and fixed with 4% paraformaldehyde, paraffin-embedded, sectioned, and stained with Masson’s trichrome stain. Stained sections were photographed using a Keyence BZ-X800 All-in-one Fluorescence Microscope, and the fibrotic area was quantified using ImageJ (NIH) (50). The proportion of fibrotic area was calculated as a ratio of the fibrotic area to the total area.

### Echocardiography.

Transthoracic echocardiography was performed using a Visual Sonics Vevo 3100 ultrasonograph outfitted with an MX400 transducer, ~30 MHz, as detailed previously (51). For acquisition of 2-dimensional guided M-mode images at the tips of papillary muscles and Doppler studies, mice were sedated by face mask administration of 1% isoflurane, hair was removed, and mice were maintained on a heated platform.

End diastolic and systolic LV diameter, as well as anterior and posterior wall thickness, were measured online from M-mode images obtained in a parasternal long axis view using the leading edge–to–leading edge convention. All parameters were measured over at least 3 consecutive cardiac cycles and averaged. LV FS was calculated as [(LV diameter_diastole_ – LV diameter_systole_)/LV diameter_diastole_] × 100; EF as [(7.0/(2.4 + LV diameter_diastole_) (LV diameter_diastole_)^3^ – (7.0/(2.4 + LV diameter_systole_) (LV diameter_systole_)^3^/(7.0/(2.4 + LV diameter_diastole_) (LV diameter_diastole_)^3^] × 100; and LV mass by using the formula 1.05 × ([posterior wall_diastole_ + anterior wall_diastole_+ LV diameter_diastole_]^3^ – [LV diameter_diastole_]^3^). Heart rate was determined from at least 3 consecutive intervals from the pulse wave Doppler tracings of the LV outflow tract. The same person obtained all images and measures.

### IHC.

Paraffin-embedded sections of heart were deparaffinized and rehydrated by washing twice with pure xylene; once with 50:50 xylene/ethanol; twice with pure ethanol; once each with 90%, 80%, and 70% ethanol in water; and once by a final wash with Milli-Q water (MilliporeSigma). Subsequently, sections were soaked in 0.5% Triton X-100 (catalog X100; MilliporeSigma) for 10 minutes to permeabilize and then rinsed 3 times with Milli-Q water. Antigen retrieval was performed by soaking in Tris-EDTA buffer (10 mM Tris, 1 mM EDTA, 0.05% Tween 20, pH 9.0) and boiling at approximately 98°C for 20 minutes. Slides were cooled to room temperature (RT), blocked by incubating in blocking buffer [PBS containing 5% goat serum (catalog G6767; MilliporeSigma), 0.1% Triton X-100 (catalog X100; MilliporeSigma), and 0.05% Tween 20 (catalog P20370-0.5; Research Products International)] at RT for 1 hour, and then incubated with primary antibodies in blocking buffer [1:400 homemade rabbit anti-RBM20 (21) or 1:500 anti–α-actinin (catalog A7811; MilliporeSigma)] overnight at 4°C. Next, slides were washed with TBS–Tween 20 (TBST) and incubated with secondary antibodies in blocking buffer [goat anti-rabbit (catalog A32731; Invitrogen) or goat anti-mouse (catalog 8890; Cell Signaling Technology)] for 1 hour at RT, washed with TBST, and mounted in SlowFade Gold Antifade Mountant with DAPI (catalog S36938; Invitrogen). Slides were photographed using a BZ-X800 All-in-one Fluorescence Microscope.

### Titin gel electrophoresis.

Titin isoforms were resolved as previously described using a 1% vertical sodium dodecyl sulfate (SDS)-agarose gel electrophoresis system (52, 53). Frozen LV myocardium from 2-month-old mice was homogenized in urea-thiourea-SDS-dithiothreitol sample buffer using a 2010 Geno/Grinder (SPEX SamplePrep) with 5 cycles of 1,500 strokes/min for 1 minute and 15-second rest between cycles (repeated 4 times for a total of 20 cycles) and subsequently incubated at 55°C for 10 minutes. The denatured protein samples were loaded on a 1% SDS-agarose gel and run at a constant current of 30 mA for 3.5 hours. The agarose gel was fixed by incubating in fixing solution (50% methanol, 12% glacial acetic acid, and 5% *w/v* glycerol) for 1 hour at RT and then dried overnight at 37°C. The dried gel was silver-stained as previously reported (52, 53) and imaged using a ChemiDoc Imaging System (Bio-Rad).

### RNA preparation and RNA-Seq.

Total RNA was extracted from the LV myocardium of 2-month-old male WT and *Rbm20*^ΔRS^ mice (*n* = 3 per group) using TRIzol Reagent (Life Technologies) and treated with DNase I (catalog D9905K; Lucigen). The concentration and purity of the isolated RNA were determined using a NanoDrop One Microvolume UV-Vis Spectrophotometer (Thermo Fisher Scientific) and electrophoresis prior to submission for RNA-Seq. RNA-Seq was performed by the University of Wisconsin-Madison Biotechnology Center Gene Expression Center & DNA Sequencing Facility. Quality check of the raw data was performed using FastQC software (54). Low-quality reads and adapter sequences were trimmed using Trimmomatic (55). Trimmed reads were mapped to the mouse reference genome (*Mus musculus* GRCm39) using STAR (56).

### Alternative splicing analysis.

Analysis of DSGs in the hearts of *Rbm20*^ΔRS^ mice relative to WT (control) was performed in Multivariate Analysis of Transcript Splicing software for replicates (rMATS) (57). Five types of alternative splicing events were analyzed, including SE, A5SS, A3SS, MXEs, and RI. The output from rMATS was filtered using FDR ≤ 5% and an absolute value of ΔPSI ≥ 10% as the cutoff criteria to identify significant alternative splicing events. Volcano plots and violin plots were plotted using the ggplot2 software package (v3.3.4) (58).

### PSI analysis.

Sequences from RNA-Seq analysis were trimmed with Trimmomatic (55) with parameters “SLIDINGWINDOW:4:15 MINLEN:36.” The trimmed sequences were then mapped to GRCm39 reference genome from Ensembl release 107 with STAR (56). PSI data were then calculated with DEXSeq (59) and PSI.sh (60) with modification adapted to Python3 and new output format of STAR. The output result was visualized using ggbio (61).

### Differentially expressed gene analysis.

For gene-level expression, gene counts were estimated using the “--quantMode GeneCounts” option in STAR (56). The R package edgeR (62) was used to normalize gene counting based on trimmed mean of M-values method (63). Only expressed genes with at least 15 counts in at least 3 samples were evaluated, resulting in 14,787 genes for further analysis. Analysis of differentially expressed genes was carried out for pairwise comparisons between *Rbm20*^ΔRS^ and WT based on a negative binomial generalized linear model using the edgeR package (62). The statistical tests were corrected for multiple testing, and only genes with an FDR less than 0.05 were considered significant (64). Enrichment network analysis was performed using Metascape (65) (http://metascape.org) under the default model. Volcano plots and heatmap were plotted using the ggplot2 software package (v3.3.4).

### RT-PCR for alternative splicing events validation.

The conditions used for the synthesis of cDNA and RT-PCR analysis have been reported previously (26). Briefly, cDNA was synthesized using iScript Reverse Transcription Supermix (catalog 1708841; Bio-Rad) following the manufacturer’s instructions. RT-PCR was carried out with EconoTaq PLUS GREEN 2X Master Mix (catalog 30033-1; Lucigen) and an initial denaturation at 94°C for 2 minutes, followed by 35 cycles of 94°C for 15 seconds, annealing for 30 seconds (see Supplemental Table 2 for primer information and anneal temperatures), and 72°C for 30 seconds. The housekeeping gene *Gapdh* was amplified by 25 cycles. PCR products were resolved on agarose gels and imaged using a ChemiDoc Imaging System (Bio-Rad).

### RT-qPCR for the validation of gene expression changes.

RNA was extracted from LV tissue (*n* = 5 for WT and *Rbm20*^ΔRS^ mice), and cDNA was synthesized as previously described (26) and described above. qPCR was performed in 384-well format using SsoAdvanced Universal SYBR Green Supermix (catalog 1725272; Bio-Rad) and a CFX384 Real-time PCR detection system (Bio-Rad). The reaction program was as follows: 95°C for 2 minutes, 39 cycles of 95°C for 15 seconds, and 60°C for 30 seconds. Finally, melting curve analysis was performed using default parameters. RT-qPCR primers are listed in Supplemental Table 2. Three technical replicates were performed for each sample. The relative amount of target mRNA (normalized to *Gapdh*) was calculated according to the 2^-ΔΔCt^ method (66).

### Western blot.

Samples were prepared for Western blot as described in the titin gel electrophoresis section above. Samples were loaded on homemade 8% polyacrylamide gels, and proteins were resolved by SDS-PAGE. Proteins were transferred to 0.2 μm Immun-Blot PVDF Membranes for Protein Blotting (catalog 1620177; Bio-Rad) at constant 30 V overnight at 4°C. Following transfer, membranes were blocked 1 hour in TBST with 10% dry milk powder (catalog DSM17200; DOT Scientific, Inc.) at RT and incubated with primary antibodies [1:500 PTBP1 (catalog 32-4800; Invitrogen) or 1:200 U2AF65 (catalog sc-166695; Santa Cruz Biotechnology)] diluted in TBST with 5% dry milk powder (catalog DSM17200; DOT Scientific, Inc.) overnight at 4°C. The next day membranes were rinsed 5 times for 5 minutes with TBST and incubated in HRP-conjugated 1:1,000 anti-mouse secondary antibody (catalog 7076; Cell Signaling Technology) diluted in TBST with 3% dry milk powder (catalog DSM17200, DOT Scientific, Inc.) for 1 hour at RT. Subsequently, membranes were rinsed 5 × 5 minutes with TBST, briefly incubated in SuperSignal West Pico PLUS Chemiluminescent Substrate (catalog 34577; Thermo Fisher Scientific), and imaged using a ChemiDoc Imaging System (Bio-Rad). After imaging, HRP was quenched by incubating in 30% hydrogen peroxide (catalog BDH7690-1; VWR International) for 30 minutes at RT. Membranes were blocked as above and incubated with rabbit anti-GAPDH primary antibody (1:1,000, catalog 5174; Cell Signaling Technology) overnight at 4°C. Membranes were rinsed and incubated in HRP-conjugated 1:3,000 anti-rabbit secondary antibody (catalog W401B; Promega) diluted in TBST with 3% dry milk powder (catalog DSM17200, DOT Scientific, Inc.) for 1 hour at RT. Membranes were rinsed and imaged as described above. Band densities were determined using ImageJ (50). Protein expression was normalized to GAPDH.

### Plasmids.

The rat pEGFP-C1-RBM20 WT mutation and deletion vectors were based on rat *Rbm20* coding sequence (NM_001107611.2) and were constructed by General Biosystems, Inc. The human pEGFP-C1-RBM20 WT and mutation vectors were based on human *RBM20* CDS sequence (NM_001134363.3) and were constructed by Gene Universal Inc. An 8xHis-tag was added at the C-terminus of RBM20.

### Cell culture, transfection, and ICC.

H9c2 cells were a gift from Jiandong Liu (University of North Carolina at Chapel Hill, Chapel Hill, North Carolina, USA) and were maintained at 5% CO_2_ and 37°C in HyClone DMEM with high glucose (catalog SH30022.01; Cytiva) supplemented with 10% fetal bovine serum (catalog SH30910.03HI; Cytiva), 1% penicillin/streptomycin (catalog SV30010; Cytiva), and 1% sodium pyruvate (catalog 11360070; Gibco, Thermo Fisher Scientific). Transfection was performed using FuGENE HD Transfection Reagent (catalog E2311; Promega) in accordance with the manufacturer’s instructions.

For ICC, H9c2 cells were grown on glass coverslips. Forty-eight hours posttransfection, cells were fixed with methanol for 15 minutes on ice. Following fixation, cells were blocked/permeabilized with PBS containing 5% goat serum (catalog G6767; MilliporeSigma), 0.1% Triton X-100 (catalog X100; MilliporeSigma), and 0.05% Tween 20 (catalog P20370-0.5; Research Products International) for 1 hour at RT. Subsequently, cells were incubated with homemade anti-RBM20 (1:400) primary antibody (21) in blocking buffer [PBS containing 5% goat serum (catalog G6767; MilliporeSigma), 0.1% Triton X-100 (catalog X100; MilliporeSigma), and 0.05% Tween 20 (catalog P20370-0.5; Research Products International)] overnight at 4°C. After washing, cells were incubated with Alexa Fluor–conjugated secondary antibodies (1:1,500; catalog A11036 or catalog A21037; Invitrogen) for 1 hour at RT, washed with PBS–Tween 20, and mounted in SlowFade Gold Antifade Mountant with DAPI (catalog S36938; Invitrogen). Images were taken using a BZ-X800 All-in-one Fluorescence Microscope (Keyence).

### Statistics.

All data are presented as mean ± SD. Methods related to statistical analysis are provided in the respective figure legends. *P* values less than 0.05 were considered statistically significant. Statistical analyses were carried out using GraphPad Prism 9.0 software.

### Study approval.

All procedures involving animals were carried out following the recommendations in the *Guide for the Care and Use of Laboratory Animals* published by the NIH (National Academies Press, 2011) and were approved by the Institutional Animal Care and Use Committee of the University of Wisconsin-Madison.

### Data availability.

Raw and processed data from RNA-Seq are available in the NCBI’s Gene Expression Omnibus database under the accession number GSE212799. The remaining data supporting the findings of this study are available within the article, supplement, or Supporting Data Values XLS file or are available from the corresponding author upon reasonable request.

## Author contributions

YZ, ZRG, and YL performed animal studies. YZ and YW carried out biochemical studies. PL and JS assisted with biochemical studies. NA helped with histology. CUB and JZ conducted RNA-Seq and bioinformatics analyses. TAH carried out echocardiography. HG provided *Rbm20*^ΔRRM^ mice. ZRG wrote the first manuscript draft. YZ, ZRG, WG, CUB, JZ, TAH, and HG edited the manuscript. WG conceived and supervised the study, analyzed data, and secured funding. YZ and ZRG were granted co–first authorship, with YZ appearing first due to greater contributions to data generation.

## Supplementary Material

Supplemental data

Supplemental table 4

Supplemental table 5

Supplemental video 1

Supplemental video 2

Supplemental video 3

Supporting data values

## Version 1 05/23/2023

In-Press Preview

## Version 2 07/10/2023

Electronic publication
