# Supplementary material for: Disruption of the nuclear localization signal in RBM20 is causative in dilated cardiomyopathy
Source: JCI Insight. 2023 Jul 10;8(13):e170001. doi: 10.1172/jci.insight.170001 (PMC10371347; doi:10.1172/jci.insight.170001)
Supplement: Supplemental data [file jciinsight-8-170001-s062.pdf]

SUPPLEMENTAL MATERIAL

Disruption of the nuclear localization signal in RBM20 is causative in dilated cardiomyopathy

Yanghai Zhang[1,#], Zachery R. Gregorich[1,#], Yujuan Wang[1], Camila Urbano Braz[2], Jibin Zhang[3], Yang Liu[1], Peiheng Liu[1], Jiaxi Shen[4], Nanyumuzi Aori[5,6], Timothy A. Hacker[7], Henk Granzier[8], Wei Guo[1,9,*]

[1]Department of Animal and Dairy Sciences, University of Wisconsin-Madison, Madison, WI 53706, USA

[2]Department of Animal Sciences, University of Illinois Urbana-Champaign, Urbana, IL 61801, USA

[3]Department of Anatomic Pathology, Comprehensive Cancer Center, City of Hope, Duarte, CA 91010, USA

[4]Department of Integrative Biology, University of Wisconsin-Madison, Madison, WI 53706, USA

[5]Department of Kinesiology, University of Wisconsin-Madison, Madison, WI 53706, USA

[6]Department of Psychology, University of Wisconsin-Madison, Madison, WI 53706, USA

[7]Division of Cardiovascular Medicine, Department of Medicine, University of Wisconsin School of Medicine and Public Health, Madison, WI 53706, USA

[8]Department of Cellular and Molecular Medicine, University of Arizona, Tucson, AZ 85724, USA

[9]Cardiovascular Research Center, School of Medicine and Public Health, University of Wisconsin-Madison, Madison, WI 53706, USA

* To whom correspondence should be addressed: Dr. Wei Guo, 1933 Observatory Dr., Madison, WI 53706, United States. Tel.: +1 608-263-3676; e-mail: wguo2@wisc.edu

[#]Equal contribution

Short title: Loss of RBM20 nuclear localization causes DCM

Table S1. Single-stranded donor and sgRNAs for CRISPR/Cas9 genome editing.

Name	Sequence (5'→3')
Single-stranded donor	CCATCTGGGTGATGCAGGTTACGAGCTCTGCAGAGTCTAAACCCTGTCTCTTCCCTTCCTCCCAGGTATGGTCCAtatGCGTGGAGAGACGAGGATCGAGAGACTGTCCCCAGGAGGGAGAACGGGGAAGACAAAAGAGACAGGTTGGATGTT
sgRNA 1	ATGGCCGTGACTCCTACGCG
sgRNA 2	CTCCCAGGTATGGTCCAGAG

Table S2. Primer information.

Experiment	Gene	Sequences (5'→3')	Annealing temp.	Products (bp)
Genotyping	*Rbm20*	F: TTCCTGGACACTCTGCACTAC<br>R: CTCATCCAACTCAGCTTTGTCC	63 ºC	WT: 405<br>ΔRS: 303
RT-PCR	*Camk2d*	F: CGAGAAATTTTTCAGCAGCC<br>R: GTCTTCATCCTCAATGGTGGTG	60 ºC	197, 155, 128, 95
RT-PCR	*Ryr2*	F: GTTGTCACGATGAAGAAGACGATG<br>R: CTTTGCTGGCACTGATAGTCTG	60 ºC	177, 153
RT-PCR	*Tpm2*	F: AGAGCCGAGGTGGCTGA<br>R: TCAGCTTCTCCTCCAGAAGT	60 ºC	154, 230
RT-PCR	*Gapdh*	F: GGTGGACCTCATGGCCTACA<br>R: CTCTCTTGCTCAGTGTCCTTGCT	60 ºC	82
RT-qPCR	*Nppa*	F: GCTTCCAGGCCATATTGGAG<br>R: GGGGGCATGACCTCATCTT	60 ºC	126
RT-qPCR	*Kcne1*	F: CTGCCCAATTCCACGACTGTT<br>R: GAGCTGAGACTTACGAGCCA	60 ºC	102
RT-qPCR	*Scn4b*	F: GGCAGATACACCTGCTTCGT<br>R: GAGTCACCGTGTTGTCCACT	60 ºC	112
RT-qPCR	*Ankrd1*	F: ATAAACGGACGGCACTCCAC<br>R: CATCTGCGTTTCCTCCACGA	60 ºC	147
RT-qPCR	*Ccn2*	F: GCCTACCGACTGGAAGACAC<br>R: GTAACTCGGGTGGAGATGCC	60 ºC	125
RT-qPCR	*Fah*	F: ACGGATTGGTGTAGCCATCG<br>R: GCTTGACCCAGACCCATGAA	60 ºC	138
RT-qPCR	*Wnt5a*	F: AGGGTTCCTATGAGAGCGCA<br>R: GCCAGACACTCCATGACACT	60 ºC	113
RT-qPCR	*Fbn2*	F: AGATGGATTCTGTTCCCGCC<br>R: TTCATGCACCTCACGCTACA	60 ºC	105

RT-qPCR	*Gapdh*	F: GGTGGACCTCATGGCCTACA<br>R: CTCTCTTGCTCAGTGTCCTTGCT	60 ºC	82

‘F’ and ‘R’ denote forward and reverse primers, respectively. ‘ΔRS’ indicates the band size in gene edited *Rbm20*$^{\Delta RS}$ mice lacking 102 bp stretch in the *Rbm20* gene corresponding to the RS domain.

Table S3. Cardiac structure and function in 4-month-old male WT (n=8) and *Rbm20*$^{\Delta RS}$ (n=7) mice, as well as female WT (n=7) and *Rbm20*$^{\Delta RS}$ (n=11) mice as assessed by M-mode echocardiography.

		**Male**			**Female**			**Two-way ANOVA (*p*-value)**		
**Parameter**	**Units**	**WT**	**ΔRS**	**Adj. *p*-value**	**WT**	**ΔRS**	**Adj. *p*-value**	**Sex**	**Genotype**	**Interaction**
Heart Rate	BPM	474.75 ± 50.04	536.30 ± 81.36	0.1787	512.72 ± 43.95	535.88 ± 71.81	0.7407	0.4462	0.0920	0.4360
LVID;s	mm	3.27 ± 0.31	4.08 ± 0.19	<0.0001	2.88 ± 0.29	3.85 ± 0.32	<0.0001	0.0098	<0.0001	0.4752
LVID;d	mm	4.29 ± 0.29	4.77 ± 0.11	0.0008	3.89 ± 0.20	4.45 ± 0.21	<0.0001	0.0002	<0.0001	0.6510
ESV	uL	43.67 ± 10.46	73.59 ± 8.32	<0.0001	32.36 ± 8.68	64.81 ± 12.28	<0.0001	0.0157	<0.0001	0.7496
EDV	uL	83.06 ± 13.34	105.87 ± 5.79	0.0006	65.99 ± 8.51	90.36 ± 10.14	0.0001	0.0002	<0.0001	0.8374
Stroke Volume	uL	39.39 ± 4.55	32.28 ± 7.50	0.0329	33.63± 2.52	25.56 ± 4.70	0.0088	0.0028	0.0004	0.8037
Ejection Fraction	%	47.92 ± 4.39	30.47 ± 6.85	<0.0001	51.65 ± 6.33	28.84 ± 7.48	<0.0001	0.6709	<0.0001	0.2800
Fractional Shortening	%	23.93 ± 2.50	14.44 ± 3.64	<0.0001	26.11 ± 3.72	13.50 ± 3.91	<0.0001	0.6446	<0.0001	0.2493
Cardiac Output	mL/min	18.70 ± 2.91	16.99 ± 3.13	0.3825	17.27 ± 2.15	13.44 ± 1.61	0.0098	0.0114	0.0054	0.2598
LV Mass	mg	91.56 ± 14.88	107.41 ± 11.11	0.0432	64.05 ± 5.43	76.29 ± 12.76	0.1061	<0.0001	0.0039	0.6897
LVAW;s	mm	0.75 ± 0.05	0.70 ± 0.05	0.3032	0.72 ± 0.09	0.60 ± 0.03	0.0011	0.0084	0.0009	0.1189
LVAW;d	mm	0.58 ± 0.05	0.60 ± 0.04	0.8457	0.54 ± 0.09	0.51 ± 0.06	0.5782	0.0065	0.7906	0.3127
LVPW;s	mm	0.77 ± 0.10	0.70 ± 0.08	0.2589	0.71 ± 0.08	0.58 ± 0.07	0.0097	0.0069	0.0034	0.3395
LVPW;d	mm	0.62 ± 0.09	0.58 ± 0.07	0.5075	0.50 ± 0.07	0.47 ± 0.06	0.6518	0.0001	0.1891	0.8403

Data are provided as mean ± standard deviation. Two-way ANOVA with the Šídák method for multiple comparisons was performed to analyze the effect of sex and genotype on each of the aforementioned parameters. Adjusted *p*-values are from the Šídák test for multiple comparisons. LVID;s, LV inner diameter during systole; LVID;d, LV inner diameter during diastole; ESV, end systolic volume; EDV, end diastolic volume; EF, ejection fraction; FS, fractional shortening; LVAW;s, LV anterior wall thickness at end of systole; LVPW;s, LV posterior wall thickness at end of systole. Data presented as mean ± SD. Two-way ANOVA with the Šídák method for multiple comparisons was performed to analyze the effect of sex and genotype on each individual parameter.

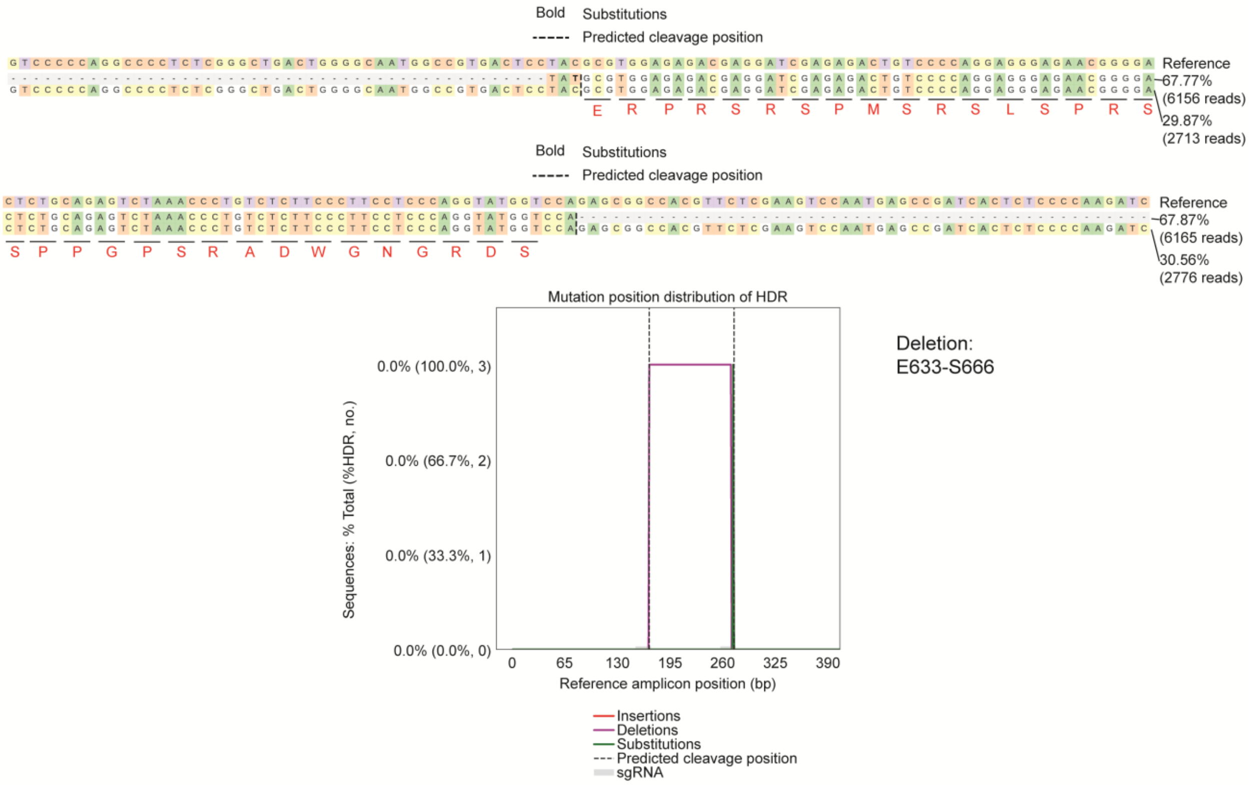

Figure S1. Confirmation of CRISPR/Cas9 genome edits in heterozygous founder via deep sequencing using an Illumina MiSeq System. The amino acid sequence of the 102 bp stretch deleted in the gene edited allele is labeled below the corresponding DNA sequence.

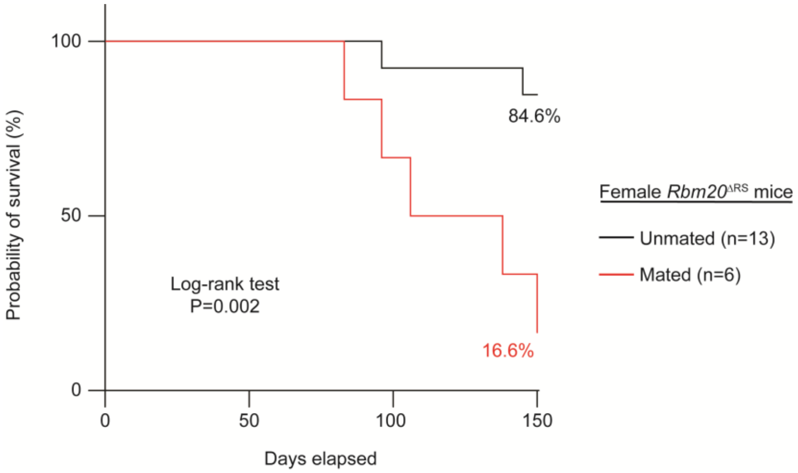

Figure S2. Survival of mated and unmated female *Rbm20*$^{\Delta RS}$ mice. The log-rank test was used to evaluate the statistical significance of differences.

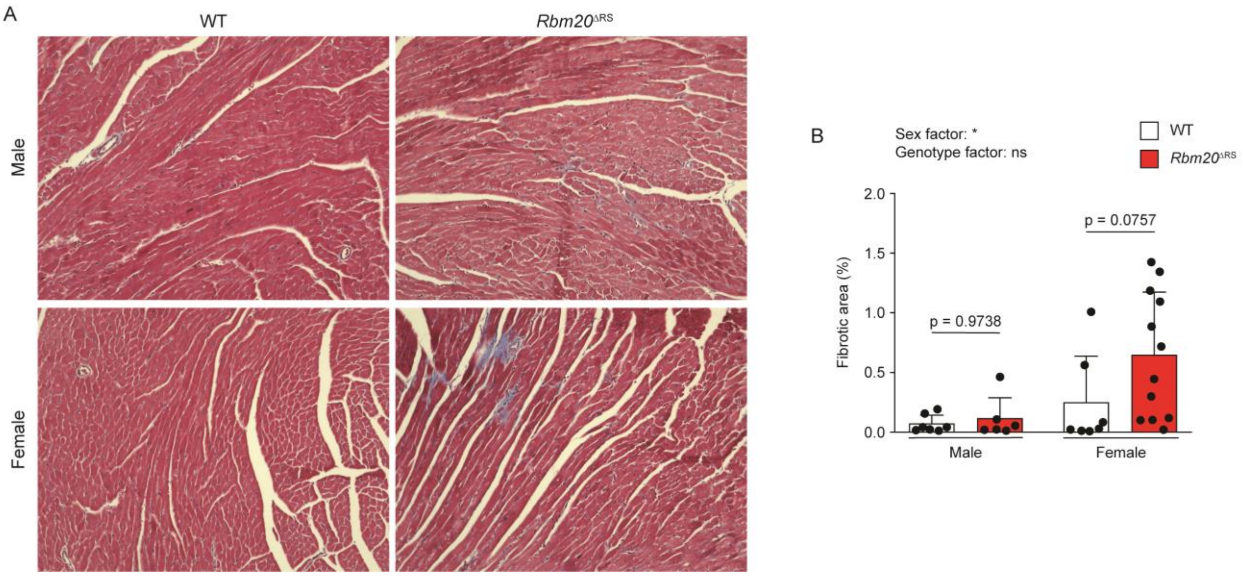

Figure S3. Quantification of fibrosis in the hearts of 4-month-old male WT (n=7) and *Rbm20*$^{\Delta RS}$ (n=6) mice, as well as female WT (n=7) and *Rbm20*$^{\Delta RS}$ (n=12) mice. A, Representative images showing Masson's trichromed stained tissue sections from male and female WT and *Rbm20*$^{\Delta RS}$ mice. Sections from at least three animals per sex and genotype were analyzed. **B**, Quantification of the fibrotic area in the hearts of WT and *Rbm20*$^{\Delta RS}$ mice of both sexes. Data presented as mean ± SD. Dots indicate measurements from individual animals. Two-way ANOVA with the Šídák method for multiple comparisons was performed to analyze the effect of sex and genotype on each individual parameter. ns, not significant; *$p<0.05$.

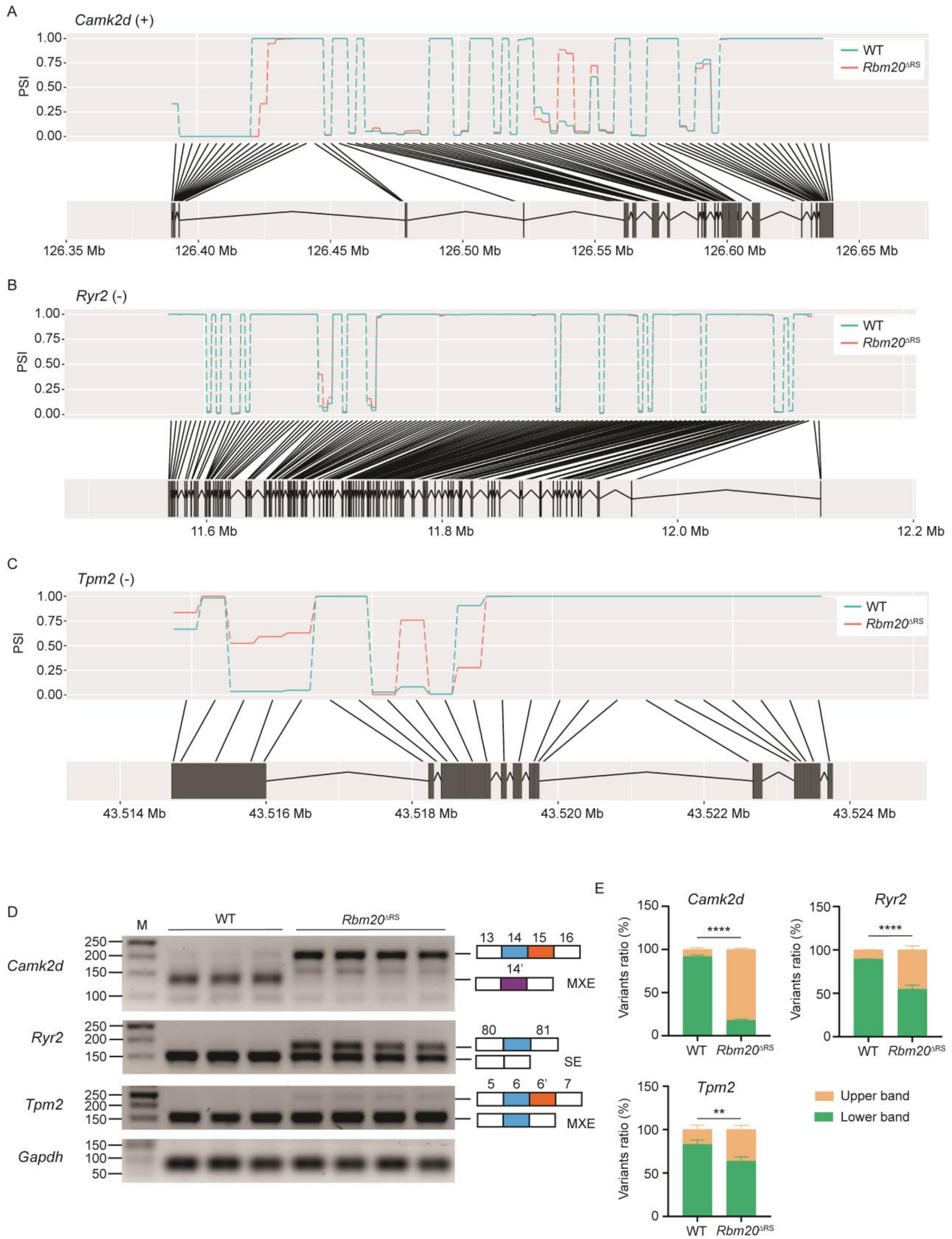

Figure S4. Validation of RBM20 target transcript splicing changes in the hearts of 2-month-old male *Rbm20*$^{\Delta RS}$ mice. A-C, RNA-seq PSI alternative splicing maps for *Camk2d* (**A**), *Ryr2*

(**B**), and *Tpm2* (**C**) comparing WT (blue) and *Rbm20*$^{\Delta RS}$ (red). **D**, RT-PCR validation of *Camk2d*, *Ryr2*, and *Tpm2* alternative splicing in the hearts of *Rbm20*$^{\Delta RS}$ mice. **E**, Quantitative analysis of RT-PCR results. The variant ratio was calculated as the intensity of the upper band versus the intensity of the lower band for each gene. Data are presented as mean ± SD. To evaluate the statistical significance of RT-qPCR results, two-tailed Student's *t*-test was performed. ns, not significant; *p<0.05; **p<0.01; ***p<0.001; ****p<0.0001.

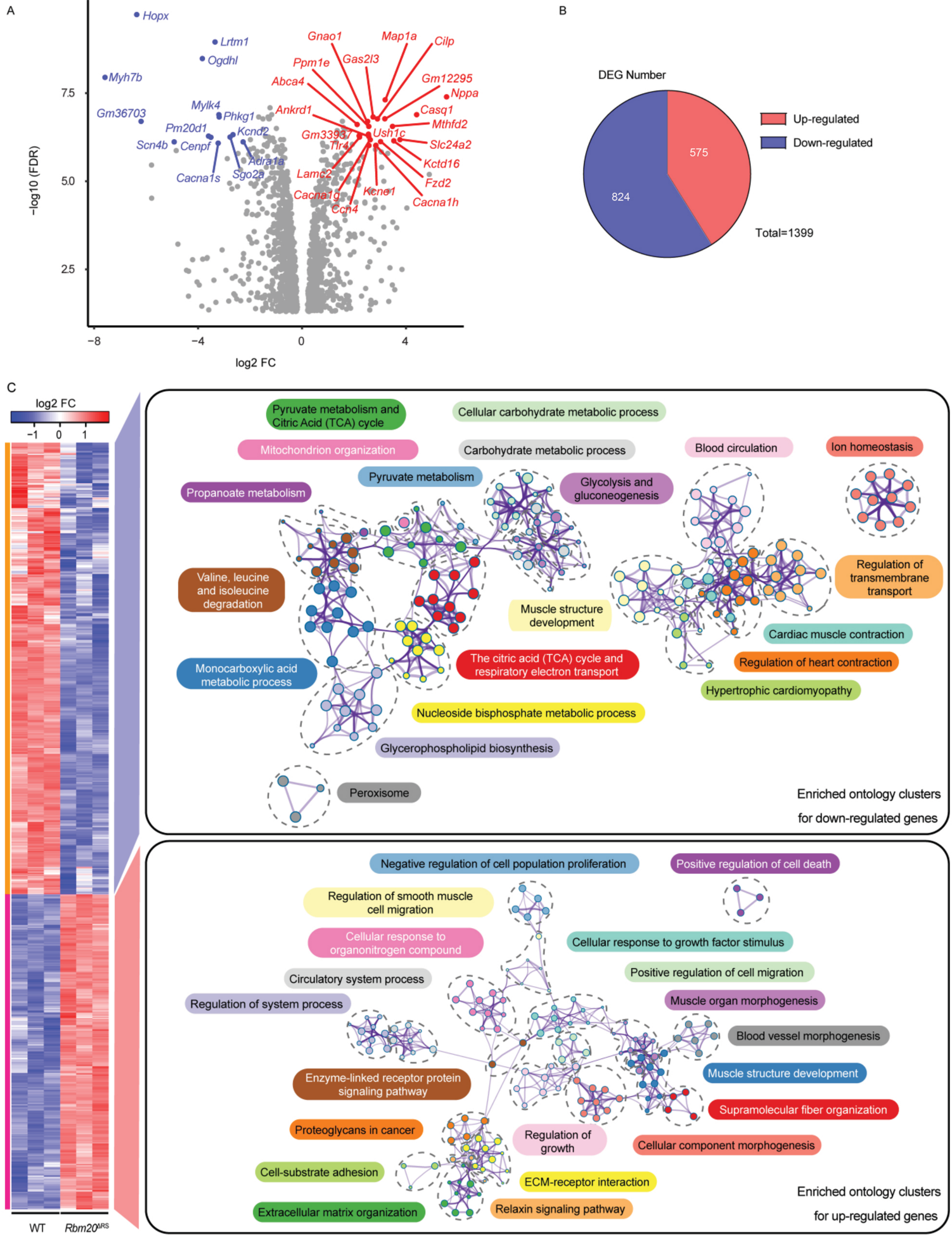

Figure S5. Analysis of genes differentially expressed in the hearts of 2-month-old male *Rbm20*$^{\Delta RS}$ mice relative to age- and sex-matched WT controls. A, Volcano plot showing genes

differentially expressed in the hearts of *Rbm20*$^{\Delta RS}$ relative to WT control mice. Log2 fold change (log2FC) was plotted against the -log10(FDR) value. Genes with -log10(FDR) > 6 and |log2FC| > 2 are indicated in red and blue, respectively. **B**, Pie chart showing the total number of up- and down-regulated genes in *Rbm20*$^{\Delta RS}$ mouse hearts versus WT. **C**, Heatmaps comparing gene expression in the hearts of *Rbm20*$^{\Delta RS}$ and WT mice. Genes down- or up-regulated in *Rbm20*$^{\Delta RS}$ mice versus WT were analyzed using Metascape and the top 20 enriched ontology clusters are shown with their representative enriched terms listed (one per cluster).

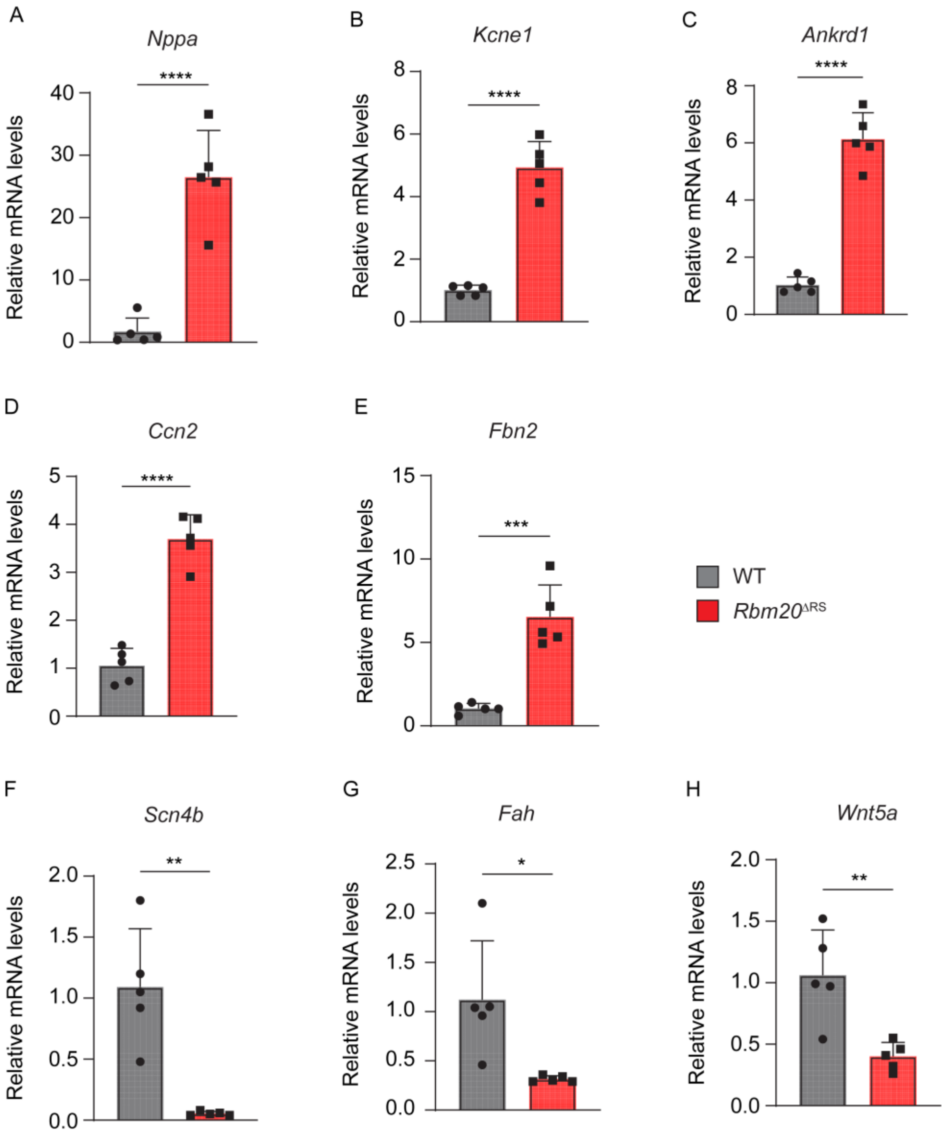

Figure S6. Validation of target gene expression changes in the hearts of *Rbm20*$^{\Delta RS}$ mice. Up-regulation of *Nppa* (**A**), *Kcne1* (**B**), *Ankrd1* (**C**), *Ccn2* (**D**), and *Fbn2* (**E**) and down-regulation of *Scn4b* (**F**), *Fah* (**G**), and *Wnt5a* (**H**) in the hearts of *Rbm20*$^{\Delta RS}$ mouse hearts was confirmed with RT-qPCR. Data are presented as mean ± SD. Dots and squares represent measurements from individual WT and *Rbm20*$^{\Delta RS}$ animals, respectively. n=5 for both WT and *Rbm20*$^{\Delta RS}$ mice. To evaluate the statistical significance of RT-qPCR results, two-tailed Student's *t*-test was performed. *$p<0.05$; **$p<0.01$; ***$p<0.001$; ****$p<0.0001$.

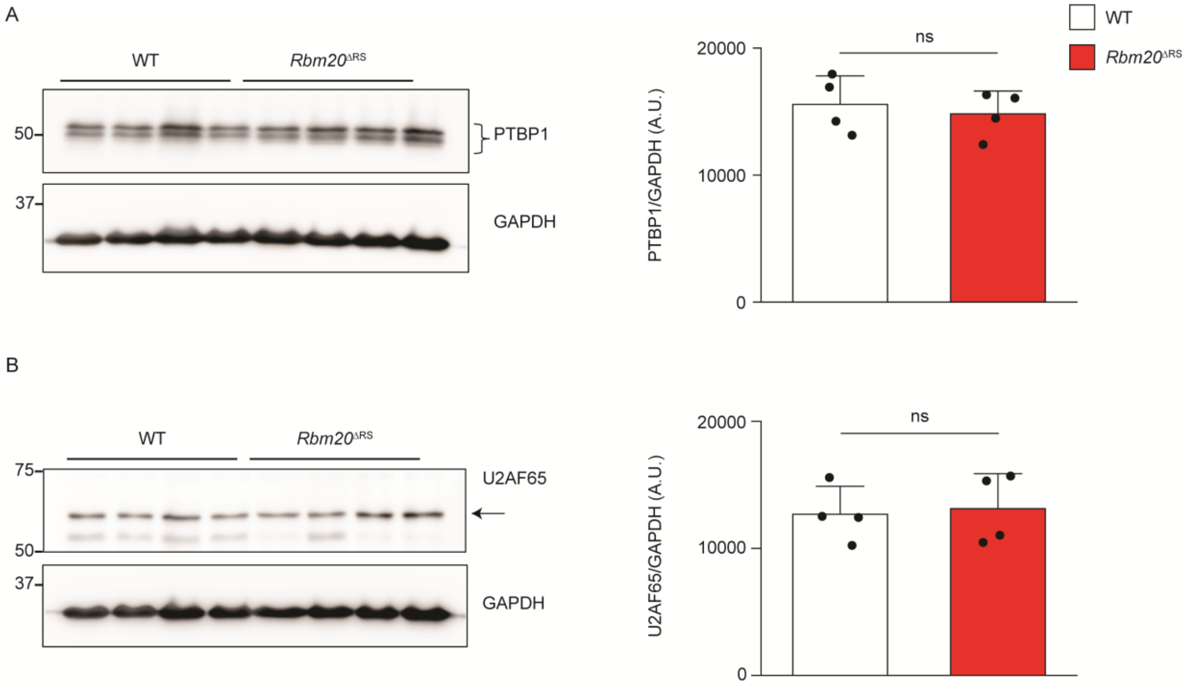

Figure S7. Western blot analysis of the expression of putative RBM20 binding partners in the myocardium of male *Rbm20*$^{\Delta RS}$ mice. A, Western blot showing expression of PTBP1 in the myocardium of WT and *Rbm20*$^{\Delta RS}$ mice (left) with quantification (right). **B**, Western blot showing expression of U2AF65 in the myocardium of WT and *Rbm20*$^{\Delta RS}$ mice (left) with quantification (right). All data are shown as mean ± standard deviation. Dots represent measurements from individual animals. n=4 for both WT and *Rbm20*$^{\Delta RS}$ mice. Two-tailed Student's *t*-test was performed to determine whether the difference between means was significant. ns, not significant.
